# Early events in dengue virus infection inducing cytokine storm: The dynamic interplay of pattern-recognition receptors, inflammasome activation, and biphasic NF-**κ**B and STAT1-dependent inflammatory responses in human mononuclear phagocytes

**DOI:** 10.1371/journal.pntd.0013366

**Published:** 2025-09-11

**Authors:** Juan Felipe Valdés-López, Yordi Sebastián Tamayo-Molina, Geysson J. Fernandez, Lady Johana Hernández-Sarmiento, Paula A. Velilla, Silvio Urcuqui-Inchima

**Affiliations:** Grupo Inmunovirología, Facultad de Medicina, UdeA, Medellín, Colombia; Linköping University: Linkopings universitet, SWEDEN

## Abstract

A Cytokine storm is critical in severe dengue, significantly contributing to disrupted endothelial integrity, plasma leakage, and haemorrhage manifestations in affected patients. Various reports have demonstrated that mononuclear phagocytes, including monocytes, dendritic cells, and macrophages, are target cells of DENV infection. They contribute to viral spread into tissues and promote robust inflammatory responses and immunopathology. However, it remains unclear whether the early events of DENV infection play a role in triggering cytokine storms in infected mononuclear phagocytes. To address this knowledge gap, we conducted a comprehensive analysis of the transcriptomic profile of *in vitro* DENV-2-infected human monocyte-derived macrophages (MDMs) based on the kinetics of viral replication through a standard growth curve. To verify the accuracy of our approach, we used RT-qPCR, ELISA, and transcriptomic data from *in vitro* DENV-2-infected monocyte-derived dendritic cells (MDDCs) and monocytes obtained from acute dengue patients. RNA-Seq analysis revealed dynamic changes in the transcriptional profile of DENV-2-infected MDMs throughout the viral growth curve. Two waves of differentially expressed genes were observed: the first occurred during the eclipse period of viral replication (3 to 5.5 h.p.i) and was associated with the induction of NF-kB-dependent pro-inflammatory factors, while the second wave at 24 h.p.i coincided with peaks of DENV-2 replication and induction of both NF-kB- and STAT1-dependent pro-inflammatory responses. Additionally, DENV-2 infection promoted the dynamic activation of Toll-like receptors, RIG-like receptors, inflammasomes, and inflammatory pathways, triggering innate pro-inflammatory and antiviral responses. A robust multi-type IFN-dependent antiviral response was also observed at the late stage of infection. A similar transcriptomic profile was found in DENV-2-infected MDDCs and monocyte subsets from acute dengue patients, further confirming the reliability of our *in vitro* model of DENV-infected MDMs. Together, results suggest that recognizing viral PAMPs during the eclipse period of DENV-2 infection promotes a robust NF-kB-dependent pro-inflammatory response in MDMs. In addition, at later stages of infection, recognizing structural DENV-PAMPs and/or viral replication intermediates induces both NF-kB- and STAT1-dependent pro-inflammatory responses, leading to a cytokine storm. These findings highlight the critical role of monocytes, macrophages, and dendritic cells in detecting DENV infection and triggering a cytokine storm *in vitro* and *in vivo*. This suggests that these cell populations could be potential targets for future immunotherapies to modulate the inflammatory response to DENV infection.

## Author summary

Dengue virus (DENV) infection can elicit an excessive and pathological immune response known as a “cytokine storm,” which plays a central role in the pathogenesis of severe dengue, leading to vascular leakage, haemorrhage, and potentially fatal outcomes. Although monocytes, macrophages, and dendritic cells are recognized contributors to this hyperinflammation, the early events that initiate cytokine storm development remain poorly defined. Here, we investigated the temporal dynamics of DENV-2-induced inflammatory responses in human macrophages by analysing transcriptomic changes throughout infection. We identified two distinct waves of immune activation: an early phase (3–5.5 hours post-infection) initiated by viral components before productive replication, and a later, more robust inflammatory response (24 hours post-infection) coinciding with peak viral replication. Both phases engaged canonical immune signalling pathways, including NF-κB and IFN-STAT1, as well as pattern recognition receptors such as Toll-like and RIG-I-like receptors. These transcriptional signatures were recapitulated in DENV-2-infected dendritic cells, and monocytes from dengue patients, underscoring their physiological relevance. Our work reveals how DENV leverages host immune sensing mechanisms to drive pathogenic inflammation. By delineating early molecular triggers, we identify potential therapeutic targets to mitigate cytokine storms and improve clinical outcomes in severe dengue.

## Introduction

With an uncontrolled increase in deforestation and the growth of human populations in urban and rural areas, global warming has converted different vector-borne agents into the most critical causes of emerging infectious diseases in the world [1,2]. Dengue fever outbreaks are caused by the four serotypes of the mosquito-borne dengue virus (DENV-1 to DENV-4), constituting one of the major public health problems in developing countries located in tropical and subtropical regions around the world [3,4]. However, due to the increase in the Earth’s average temperature caused by global warming, the geographic distribution and adaptation of the viral vector mosquitoes *Aedes* (*Ae*.) *aegypti* and *Ae. Albopictus* has expanded, making dengue a critical health problem in regions previously considered inaccessible to the virus, including Western Europe, North America, and Central and North Asia [5]. According to the Pan American Health Organization (PAHO), between epidemiological weeks 1 and 50 of 2024, a total of 12,912,635 suspected dengue cases were reported in the Americas, with 849,856 cases confirmed by laboratory, and 22,284 cases were classified as severe dengue. A total of 8,046 deaths from dengue patients were recorded, for a fatality rate of 0.062%, and a cumulative incidence of 1,352 dengue cases per 100,000 population, which represents an increase of 289% compared to the same period in 2023, and 364% compared to the average of the last 5 years [6].

The clinical manifestation of the infection by any DENV serotypes can span a broad spectrum of outcomes, and according to the PAHO, it clinically divides cases into dengue, dengue with warning signs (DWS), and severe dengue (SD) [7]. Dengue is characterized by clinical features such as fever, headache, retro-orbital pain, leukopenia, nausea/vomiting, rash, myalgia, and/or arthralgia [7]. However, the progression of dengue to DWS/SD is marked by a pathological immune response characterized by systemic overproduction of inflammatory cytokines and chemokines leading to cytokine storm, which is considered a key factor in dengue immunopathogenesis by promoting endothelial hyperactivation, disruption of endothelial-integrity, plasma leakage, multiple organ failure, and death [7–12]. To date, predicting the disease outcome in dengue infections remains impossible, and no specific antiviral treatment is currently available. In addition, the only approved vaccines, Sanofi Pasteur’s Denvaxia and Takeda’s Qdenga (Tak-003), offer partial protection and have limitations in their efficacy [13].

DENV is a member of the *Flavivirus genus* within the *Flavivirus family* [4]. To initiate infection, DENV binds to a broad array of receptors on the host cell surface, including the macrophage mannose receptor (MMR/CD206) [14], dendritic cell-specific intercellular adhesion molecule-3-grabbing non-integrin (DC-SIGN) [15], low-density lipoprotein receptor-related protein-1 (LRP1) [16], C-type lectin domain family 5 member A (CLEC5A) [17], and Toll-like receptor 2 (TLR2), along its co-receptor CD14 [18]. Then, viral particles are internalized via clathrin-mediated endocytosis [19]. Subsequent acidification of the endosome triggers conformational changes in the viral Envelope (E) protein, leading to the fusion of the viral and endosomal membranes and the release of viral nucleocapsid into the cytoplasm [20]. The viral genome, a single-stranded positive-sense RNA (ssRNA+), is then released into the cytoplasm. The viral genome encodes a single polyprotein, which is cleaved by viral and host proteases into structural (C, prM, E) and nonstructural (NS1, NS2A/B, NS3, NS4A/B, NS5) proteins [21]. NS5, an RNA-dependent RNA polymerase, synthesizes a complementary negative-sense RNA strand, which serves as a template for producing new DENV ssRNAs and double-stranded RNAs (dsRNA) [21–23]. The structural proteins and RNA genome assemble into immature virions within the endoplasmic reticulum [24,25]. Finally, exocytosis releases mature and immature DENV particles from infected cells [25].

Mononuclear phagocytes, including monocytes, macrophages, and myeloid dendritic cells, can replicate DENV and sense the infection through an array of pattern-recognition receptors (PRRs) involved in recognizing pathogen-associated molecular patterns (PAMPs) present in DENV particles and infected cells [18,26,27]. Previous studies have identified various PRRs involved in recognizing DENV proteins or replication intermediates. These include TLRs such as TLR2/CD14 (E protein) [18], TLR3 (dsRNA) [28], TLR4 (NS1 protein) [29], TLR7/8 (endosomal ssRNA) [30,31]; NOD-like receptors (NLRs) including NOD2 [32], and NLRP3 (M protein) [33]; and viral RNA sensors including retinoic acid-inducible gene-I (RIG-I) (5’-structure in ssRNA) [34]; interferon-induced protein with tetratricopeptide repeats (IFIT)-family proteins (ssRNA) [35], and dsRNA-activated protein kinase (PKR) (dsRNA) [36]. The recognition of DENV structural proteins or replication intermediates by PRRs ultimately activates signalling pathway that triggers innate inflammatory and antiviral responses to DENV, aiming to control viral replication and spread [32,37,38]. This response involves various immune cells and inflammatory mediators that contribute to the induction of cytokine and chemokine release, but also interferons (IFNs), which play a crucial role in containing the infection [9,10,18,33–39]. Importantly, however, the molecular mechanism underlying the imbalance in the inflammatory response that exacerbates the immune response and increases dengue severity, both *in vitro* and *in vivo*, is not fully understood. Likewise, the spectrum of cytokines and chemokines involved in the induction of cytokine storm by DENV-infected mononuclear phagocytes remains unknown. Therefore, to address this knowledge gap, we analysed the transcriptomic profile of human monocyte-derived macrophages (MDMs) infected with DENV-2 *in vitro* based on the kinetics of viral replication through a standard growth curve, and the transcriptomic profile of DENV-2-infected monocyte-derived dendritic cells (MDDCs), and monocyte subsets obtained from acute dengue patients.

## Materials and methods

### Ethics statement

The study was approved by the Ethics Committee of the “Sede de Investigación Universitaria-Universidad de Antioquia.” Written informed consent was obtained from all individuals who voluntarily participated in this study according to the principles of the Declaration of Helsinki. Four healthy donors were included in this study.

### DENV-2 stock and viral titration

The highly pathogenic DENV-2 New Guinea C (NGC) strain, characterized by its ability to induce high viremia and severe infection in non-human primates [40], was propagated in *Ae. Albopictus-*derived C6/36-HT cells (ATCC) using a multiplicity of infection (MOI) of 0.01. The C6/36 HT were grown in Leibovitz’s L-15 medium (L-15; Sigma-Aldrich) supplemented with 5% heat-inactivated fetal bovine serum (FBS; Gibco, Thermo Fisher Scientific, Massachusetts, USA) and 1% antibiotic-antimycotic solution (Corning, New York, USA), and incubated at 34°C. DENV-2 culture supernatants were stored at −80°C and titrated by plaque assay on BHK-21 cells (clone 15, ATCC), as previously reported [41].

### Cultures of primary human monocytes and differentiation into monocyte-derived macrophages

Human peripheral blood mononuclear cells (PBMCs) were isolated from leukocyte-enriched blood units from healthy donors (n = 2–4) through a density gradient with Lymphoprep (STEMCELL Technologies Inc., Vancouver, Canada) by centrifugation at 850 x g for 21 min. Platelet depletion was performed by washing with Phosphate-buffered saline (PBS) (Sigma-Aldrich) three times at 250 x g for 10 min, and the percentage of CD14 + positive cells was determined using flow cytometry. To obtain monocyte-derived macrophage (MDM), 5x10^5^ CD14-positive cells were seeded into each well of 24-well plastic plates as described in [42,43]. Briefly, the monocytes were allowed to adhere for 2 h in RPMI-1640 medium (Sigma-Aldrich) supplemented with 0.5% autologous serum, 4 mM L-glutamine, and 0.3% NaCO_3_ and cultured at 37°C and 5% CO_2_. Non-adherent cells were removed by washing twice with PBS 1X, and monocytes were cultured in RPMI-1640 medium supplemented with 10% FBS, 0.3% NaCO_3_, 1% antibiotic-antimycotic solution 100X, and were incubated at 37°C and 5% CO*2* for 6 days to obtain MDMs, fresh media was added to the cultures every 2 days.

### *In vitro* DENV-2 infection of monocyte-derived macrophages

MDMs were infected with DENV-2 at MOI 5 in serum-free RPMI-1640 and incubated at 37 C for 1.5 hours, as previously reported [41]. Then, the cells were washed with PBS to remove the unbound virus, and a fresh, complete medium was added. MDMs were incubated at 37 C and 5% CO_2_. Culture supernatants and cell lysates were collected at 1.5-, 3-, 5.5-, and 24-hour post-infection (h.p.i) and stored at −80 C.

### Flow cytometry analysis

A BD LSRFortessa flow cytometer (BD Biosciences, New Jersey, USA) was used to assess cell size (FSC-A) and cytoplasmic complexity/granularity (SSC-A) of MDMs. To quantify the expression of cell surface macrophage markers, uninfected MDMs were stained with following mouse anti-human antibodies: CD68 (PE, clone: eBioY1/82A), TLR2 (PE, clone: TL2.1), TLR4 (PE, clone: HTA125), CD14 (FITC, clone: 61D3), and MMR/CD206 (PE, clone: 19.2) (eBioscience, Massachusetts, USA, and BD Bioscience), or their respective isotypes controls. Staining was performed in PBS for 30 minutes at 4°C. The cells were then washed by centrifugation and resuspended in PBS.

DENV-2 infection in MDMs was evaluated by intracellular staining of the viral envelope (E) protein. Briefly, MDMs were harvested from culture and fixed using a fixation/permeabilization buffer (eBioscience, USA). After washing, MDMs were stained with a mouse anti-flavivirus E antibody (inhouse, clone: 4G2) for 30 minutes, followed by a goat anti-mouse IgG-PE secondary antibody (Thermo Scientific, USA) for 30 min. Positively labelled cells were defined using isotype controls and unstained cells. A compensation matrix was applied to compensate for spectral overlap. At least 20,000 events were acquired for each sample, and data were analysed using FlowJo software (version 10). Debris and dead cells were gated out using forward and side scatter gating.

### RNA extraction and Bulk RNA-seq of DENV-2-infected MDMs

Total RNA was extracted using the Direct-zol RNA Miniprep Plus (Zymo Research, California, USA) according to the manufacturer’s instructions. RNA samples were treated with DNase I (Zymo Research) to remove genomic DNA. RNA concentration was determined by spectrophotometry using a Nanodrop (Thermo Scientific, Massachusetts, USA), and 300 ng of RNA was used for bulk RNA-Seq, which was performed on an Illumina HiSeq 4000 platform. After sequencing, the image data were converted into raw reads and stored in FASTQ format for each sample, with quality assessment using FastQC (google/vFqiZ), as previously described [44]. Then, clean reads were obtained by removing low-quality adapter, poly N-containing, and shorter-than-70 bp reads. The location of the reads on the reference genome was determined quickly and precisely by comparing reads with the reference genome (GRCh38 or DENV-2 [NC_001474]) using HISAT2 software [45]. The new transcripts were then assembled using String Tie software [46], and using the feature counts tool in Subread software [47], the raw count number in each sample was obtained. Next, raw counts were subject to the following workflow executed on R software (version 4.2.0). Initially, we annotated gene rows with their respective genotype (non-coding gene, pseudogene, and protein-coding gene), symbols, and EntrezID. Based on the genotype, we selected only those protein-coding genes and then generated a list with their lengths using the Homo.sapiens library. Batch effect correction on raw count data for each transcriptome was performed using ComBat seq [48]. Then, to determine the top DEG, we selected genes with a false discovery rate (FDR) < 0.05 and |Log_2_ Fold Change (FC) (DENV-2-infected MDMs/control MDMs) > 0.6, using DESeq2 library [49]. Then, the raw counts were normalized to transcripts per million (TPM) using R statistical software (version 4.2.0). As previously reported, we selected the genes linked to JAK-STAT signaling, pattern recognition receptors (PRRs) signaling, and antiviral and inflammatory responses [42,50]. We plotted the data using bar plots and heatmaps with GraphPad Prism for Windows (GraphPad Software version 8.0.1) and R statistical software. Gene set enrichment analyses (GSEA) for overall DEG regulated in DENV-2-infected MDMs were performed using the ClusterProfiler package (Version 4.0) [51]. A gene co-expression network was constructed using Pearson correlation coefficients. The data were filtered to retain only significant correlations (p < 0.05), and only pairs involving DENV-RNA were selected. The network was visualized using ggraph with the “graphopt” layout algorithm. Edges were coloured according to the sign of the correlation (positive or negative) and weighted by the absolute value of the correlation. The Raw RNA-seq data have been deposited in GEO under accession number GSE297386.

### RNA extraction, cDNA synthesis, and real-time qPCR

Total RNA was extracted from MDMs infected or not with DENV-2 at an MOI of 5, at 1.5, 10, and 24 h.p.i. Then, 300 ng of RNA was used to construct cDNA libraries for each experimental group using the commercial RevertAid Minus First Strand cDNA Synthesis Kit (Thermo Scientific), following the manufacturer’s protocol. RT-qPCR was used to validate the expression of 19 key DEGs identified through bulk RNA-seq, using a set of specific primers (S1 Table). RT-qPCR amplifications were performed using the Maxima SyBR-Green system (Thermo Fisher Scientific). The Bio-Rad CFX manager obtained the cycle thresholds (Ct) determined for each sample using a regression fit in the linear phase of the RT-qPCR amplification curve. The relative expression of each target gene was normalized to that of the unstimulated control and housekeeping gene GAPDH.

### Cytokine quantification

Levels of tumor necrosis factor-alpha (TNF-α), interleukin 6 (IL-6), CXCL8/IL-8, and CCL-2 were quantified in MDM culture supernatants using commercially available ELISA kits (BioLegend) following the manufacturer’s instructions. The detection limit was 2–10 pg/mL.

### Transcriptomic analysis of DENV-2-infected MDDCs and monocyte subsets from acute dengue patients

To extrapolate the transcriptomic results obtained in DENV-2-infected MDMs to other *in vitro* and *in vivo* models of DENV infection, and confirm the biological relevance of biomarkers identified in DENV-2-infected MDMs, we reanalysed the publicly available Microarray dataset (**GSE58278**) [52], performed in human Monocyte-derived dendritic cells (MDDCs) infected with DENV-2 NGC, at MOI 20, for 24 hours. Also, we reanalysed the publicly available bulk RNA-seq dataset (**GSE176079**) [53], generated from sorted classical (CM, CD14^++^CD16^-^), intermediate (IM, CD14^++^CD16^+^), and non-classical (NM, CD14^Dim^CD16^+^) Mon subsets isolated from six acute dengue patients (ADP) and three healthy donors. To determine the top DEG, we selected genes with a false discovery rate (FDR) < 0.05 and |Log_2_ Fold Change (FC) (DENV-infected cells/Uninfected control) > 0.6, using GEO2R.

### Statistical analysis

Statistical analysis was performed using GraphPad Prism for Windows (GraphPad Software version 8.0.1, San Diego, CA, USA; www.graphpad.com). Shapiro-Wilks test was performed to determine the normality of the data. Data are represented as mean ± SEM or as median with interquartile range. The statistical tests are indicated in the fig legends. Significant results were defined for multiple t-tests in the multitranscript analysis p < 0.05 (*) and in the validation experiments for Kruskal Wallis or repeated measures Anova p < 0.05 (*).

## Results

### Human monocyte-derived macrophages are susceptible and permissive to DENV-2 infection and exhibit dynamic transcriptional response *in vitro*

**Fig 1:**
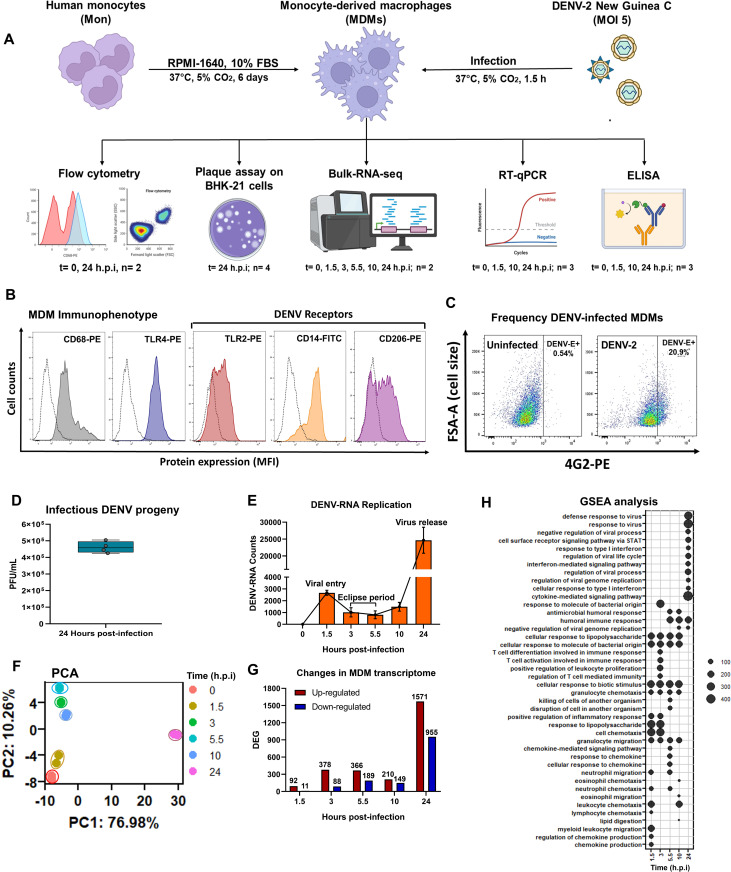
DENV-2 infection induces a dynamic transcriptional profile in human macrophages. **(A)** Schematic overview of the experimental design. The flowchart was created using BioRender. **(B)** Immunophenotype of human MDMs. **(C)** Frequency of DENV-infected MDMs at 24 h.p.i. **(D)** Production of infectious DENV-2 viral particles at 24 h.p.i. **(E)** Quantification of DENV-2 RNA in infected MDMs. **(F)** PCA analysis of transcriptomic data. **(G)** DEG, both upregulated and downregulated, in DENV-2-infected MDMs. DEGs were identified using DESeq2 with a p-value < 0.05 and |Log2 Fold Change (DENV-2-infected MDMs vs Control MDMs) | > 0.6. **(H)** Gene ontology analysis of DEGs upregulated by DENV-2 infection in human MDMs.

Cytokine storm is a key factor in endothelial-integrity disruption, plasma leakage, and haemorrhage in severe dengue patients [8,10,18,29,54]. Mononuclear phagocytes, as macrophages, are essential components of the innate immune response that play a critical role in the control and immunopathogenesis of dengue (Reviewed in [55]). Human macrophages are target cells for DENV infection, contributing to viral replication and promoting robust inflammatory responses [41,50]. However, the role of macrophages in inducing cytokine storms during the early stages of DENV infection is still poorly understood. To investigate this, human monocytes from healthy donors were isolated and differentiated into MDMs over six days in RPMI-1640 medium supplemented with 10% FBS, as previously reported [41,50]. MDMs were then infected at MOI 5 of the highly pathogenic DENV-2 NGC strain for 0 (control), 1.5, 3, 5.5, 10, or 24 hours, after which various immunological and molecular assays were performed, as outlined in Fig 1A. First, we confirmed the macrophage immunophenotype of our *in vitro* MDM model by flow cytometry. MDMs expressed high levels of classical macrophage markers, including CD68 (Macrosialin) and TLR4 (Fig 1B). In addition, they expressed high levels of DENV-entry receptors, including TLR2, CD14, and macrophage mannose receptor CD206 (Fig 1B), supporting their susceptibility to DENV infection.

Next, we evaluated the permissiveness of the MDMs model to DENV-2 replication by quantifying the frequency of infected cells by flow cytometry, and the production of infectious viral particles in culture supernatants at 24 h.p.i by plaque assay using BHK-21 cells. We found that approximately 21.95 ± 1.48% of cultured MDMs were infected and expressed DENV-E antigen (Fig 1C). Furthermore, high levels of infectious viral particles were detected at 24 h.p.i (mean 4.6x10^5^ PFU/mL; Fig 1D), confirming both the susceptibility and permissiveness of our *in vitro* MDM model to DENV-2 infection.

To examine the kinetics of DENV-2-RNA synthesis and the associated transcriptomic changes in infected MDMs, we performed bulk RNA-seq. The results show an early peak in DENV-2-RNA accumulation at 1.5 h.p.i. However, a reduction in DENV-2-RNA copy number was observed at 3 and 5.5 h.p.i, (Fig 1E), suggesting partial degradation of internalized viral particles or RNAs by MDMs. This period was defined as the eclipse period of infection, representing the interval between viral entry and the initiation of productive RNA synthesis and virion assembly. Subsequently, viral RNA synthesis increased markedly from 10 h.p.i and reached its maximum level at 24 h.p.i (Fig 1E), consistent with the timing of viral release (Fig 1D). These results are in line with our previous observations on DENV replication in human MDMs [41,50].

In line with the kinetics of viral replication, PCA analysis of host gene expression (Fig 1F) revealed dynamic changes in the MDM transcriptome, with the most pronounced alterations occurring at 24 h.p.i. Notably, PCA also indicated low inter-individual variability between MDMs derived from two independent donors, supporting that the observed transcriptional changes were driven by DENV-2 infection rather than donor-specific genetic background. Additionally, RNA-seq analysis revealed transcriptional changes during the viral growth curve of DENV-2 infection (Fig 1G). Differentially expressed genes showed two waves: the first during the viral entry-eclipse periods (1.5 to 5.5 h.p.i), with the lowest number of transcribed genes at 10 h.p.i, and a maximum peak at 24 h.p.i. A total of 92, 378, 366, 210, and 1571 DEG were upregulated by DENV-2 infection at 1.5, 3, 5.5, 10, and 24 h.p.i, respectively, while DENV-2-infected MDMs downregulated 11, 88, 189, 149, and 955 DEG at 1.5, 3, 5.5, 10, and 24 h.p.i, respectively. DEGs induced during early phase of infection (eclipse period) were associated with robust inflammatory responses (Fig 1H), showing significant enrichment of biological processes related to granulocyte recruitment and activation, as well as the induction of pro-inflammatory pathways. In contrast, at 24 h.p.i, we observed significant enrichment of pathways linked to the activation of IFN-dependent JAK-STAT signaling and establishing an antiviral state. Together, these findings suggest that human MDMs mount a dynamic and complex immune response to DENV-2 infection, characterized by a temporal shift from early inflammation to a later antiviral response.

### Active DENV-2 replication induces a differential gene expression profile of pattern-recognition receptors in human macrophages

**Fig 2:**
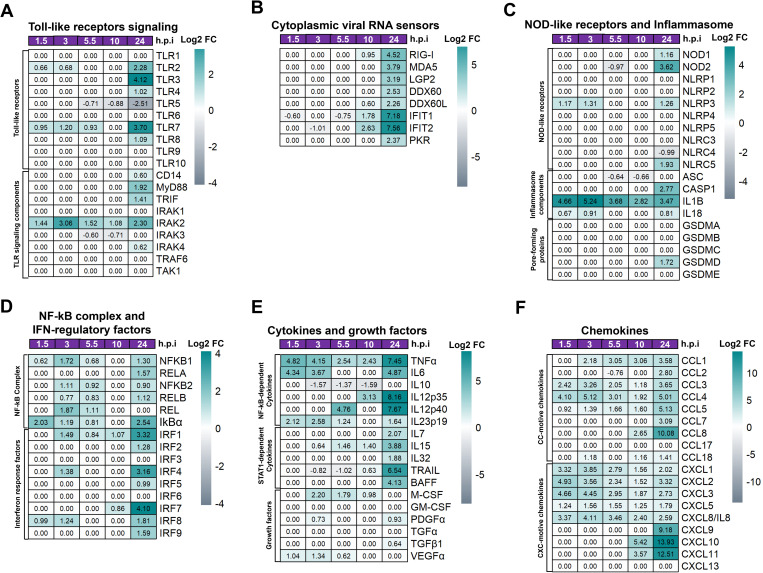
Active DENV-2 replication induces differential expression of pattern-recognition receptors and inflammatory mediators in human macrophages. Human primary MDM cultures were either infected or not with DENV-2, as outlined in Fig 1A. Gene expression (mRNA) is presented as Log2FC heatmaps. Shown are the mRNA expression profiles of: **(A)** Toll-like receptor signalling components. **(B)** cytoplasmic viral RNA sensors. **(C)** NOD-like receptors and inflammasome components. **(D)** NF-kB complex and IRFs. (E) cytokines and growth factors. (F) chemokines. DEGs were identified using DESeq2, with a p-value < 0.05, and |Log2 Fold Change (DENV-2-infected MDMs vs Control MDMs) | > 0.6.

Macrophages play a key role in the induction of innate immune antiviral response by activating different PRRs to promote the activation of signaling pathways that lead to the induction of inflammatory and antiviral factors [41,50,56,57]. As shown in Fig 2A, the expression pattern of TLRs is very dynamic and varies depending on the time of infection. While DENV-2 infection-induced up-regulation of *TLR2* and *TLR7* mRNA during the early stages of infection (1.5 and 3 h.p.i), the mRNA expression of *TLR2*, *TLR3*, *TLR4*, *TLR7*, and *TLR8* reaches its maximum peak of expression at 24 h.p.i (Figs 2A, S1A). Interestingly, *TLR5* mRNA expression was significantly downregulated starting at 5.5 h.p.i, with a peak of transcriptional repression observed at 24 h.p.i. In contrast, *TLR9* mRNA expression was not induced (Figs 2A, S1A). TLRs induce inflammation through MyD88 or TRIF adaptor molecules [42,58]. Next, we determine if DENV-2 infection promotes the expression of these components in MDMs. While *MYD88* showed a maximum expression peak at 24 h.p.i, *TRIF* exhibited two peaks: a minor peak at 1.5 h.p.i, and a maximum peak at 24 h.p.i (Figs 2A, S1A). Altogether, these results suggest that TLRs could play a crucial role in the recognition of DENV-PAMPs from the early hours of infection, with a peak response at 24 h.p.i.

Next, we evaluated the expression of various PRRs involved in recognizing nucleic acids, including viral RNA sensors in DENV-2-infected MDMs (Figs 2B, S1B). mRNA levels of viral RNA sensors, including *RIG-I*, Melanoma differentiation-associated protein 5 (*MDA5*), *IFIT1*, *PKR*, and other RNA sensors increased following DENV-2-RNAs replication in MDMs, with their expression peaking at 24 h.p.i (Figs 2B, S1B). The expression pattern of viral RNA sensors was consistent with DENV-2-RNA replication observed in infected MDMs (Fig 1E). These findings suggest that the induction of RNA sensors, like to TLRs or RIG-I, may play a key role in detecting DENV-RNAs and promoting innate immune antiviral responses in infected MDMs.

### DENV-2 infection promotes a dynamic transcriptional induction of NOD-like receptors and inflammasomes signaling in human macrophages

Previous reports showed that DENV infection promotes NLRP3-inflammasome complex activation in different cell populations to induce the proteolytic maturation of pro-IL-1β and pro-IL-18 into IL-1β and IL-18, respectively [59,60]. Both interleukins play a key role in dengue immunopathogenesis, and high IL-1β serum levels correlate with the severity of dengue in patients [10,18,33]. However, the dynamics of inflammasome transcription at early and late stages in DENV-infected macrophages are poorly understood. DENV-2 infection upregulated the expression of NLRP3-inflammasome components, including *NLRP3*, as well as *pro-IL1β* and *pro-IL18* with an initial peak of mRNA expression at 1.5 and 3 h.p.i, followed by a second peak at peak at 24 h.p.i (Figs 2C, S1C). Additionally, DENV-2 infection upregulated the mRNA expression of other NLRs, including *NOD1,* and *NLRC5*, all of which had mRNA expression peak at 24 h.p.i (Figs 2C, S1C). Next, we determined the mRNA expression of essential components of NLRP3 signaling pathway, Caspase 1 (*CASP1*) and Apoptosis-associated speck-like protein (ASC). In both infected and uninfected MDMs, high mRNA levels of *ASC* mRNA were observed, which decreased at 5.5 and 10 h.p.i, then increased again at 24 h.p.i. In contrast, *CASP1* and Gasdermin D (*GSDMD*) expression remains unchanged during the eclipse period of infection but increased significantly at 24 h.p.i (Figs 2C, S1C). Together, these results suggest that priming signals for induction of *NLRP3*, *pro-IL1β* and *pro-IL18* mRNA expression occur at early stages during the eclipse period of DENV-2 replication in MDMs. However, the induction of effector molecules involved in inflammasome activation, including *CASP1* and *GSDMD,* occurs later in the infection, coinciding with high levels of DENV-2 replication.

### DENV-2 infection promotes a dynamic transcriptional modulation of NF-kB complex and interferon-regulatory factors in human macrophages

Activation of PRRs triggers signaling pathways that lead to the activation of transcription factors, including the Nuclear factor kB (NF-kB) complex and Interferon-regulatory factors (IRFs) [42,57]. Those, in turn, promote the production of cytokines, chemokines, and/or interferons, all of which contribute to effective inflammatory and antiviral responses [42,57]. Our transcriptomic analysis showed that DENV-2 infection promotes a dynamic modulation of NF-kB-complex components, including *NF-kB1*, *NF-kB2*, *RELA*, and *I*κ*B*α** (Figs 2D, S1D). *NF-*k*B1* and *NF-*k*B2* showed a maximum expression peak during the early stage of infection (3 h.p.i), followed by a decrease to a minimum at 10 h.p.i, before increasing again at 24 h.p.i. *RELA* significantly increase its expression at 24 h.p.i (Figs 2D, S1D). In contrast, **IkB*α*, a negative regulator of NF-kB, was significantly induced early in the infection (1.5 h.p.i) and again at a later stage (24 h.p.i; (Figs 2D, S1D).

Like the NF-kB complex, DENV-2 infection promotes dynamic modulation of interferon-regulated factors (IRFs). Although *IRF1*, *IRF7*, and *IRF8* exhibited a peak of maximum expression at 24 h.p.i, *IRF1* and *IRF8* mRNA levels also increased during the first 3 h.p.i before decreasing to their lowest levels between 5.5 and 10 h.p.i (Figs 2D, S1D). Together, these results suggest that DENV-2 infection induces two expression peaks for the NF-kB-complex and IRFs in infected MDMs. This modulation may be dependent on the recognition of structural PAMPs present in DENV-2 particles during the early hours of infection or, later, the recognition of DENV-2 replication intermediates (dsRNA), viral genome (ssRNA), and/or viral proteins during the peak of active viral replication and viral release.

### DENV-2 infection of human macrophages promotes dynamic and robust NF-kB- and STAT1-dependent pro-inflammatory responses

In line with results presented in Fig 2D, DENV-2 infection promotes a dynamic transcription of NF-kB-target genes in MDMs, including Tumoral necrosis factor-α (*TNFα)*, Interleukin 6 (*IL6)*, and *IL10* (Figs 2E, S1E). *TNFα* and *IL6* exhibited two peaks of transcription, one at 1.5 h.p.i, and another at 24 h.p.i, whilst *IL10* increased slightly at 1.5 h.p.i, and then drastically decreased from 3 to 24 h.p.i, suggesting that active DENV-2 replication negatively regulated *IL10* gene transcription in infected MDMs. Furthermore, we found that DENV-2 infection induced the expression of different members of the IL-12 family of cytokines in MDMs (Figs 2E, S1E). While the IL-12p70 subunit, *IL12p35,* was not expressed during the first 10 h.p.i, its transcription reached a maximum peak at 24 h.p.i. *IL12p40*, however, showed an initial expression peak at 3 h.p.i, with a maximum expression peak at 24 h.p.i (Figs 2E, S1E). Similarly, *IL23p19*, one of the subunits of IL-23 (IL23p19/IL12p40), reached its maximum expression peak at 3 h.p.i, then decreased drastically with a lower peak at 10 h.p.i, and increased slightly at 24 h.p.i (Figs 2E, S1E). These results suggest a dynamic regulation in the induction of IL-12 family subunits during DENV-2 infection in MDMs.

Interestingly, DENV-2 infection induced mRNA expression of different growth factors in MDMs, including Macrophage colony-stimulating factor (*M-CSF*) and Vascular endothelial growth factor-α (*VEGFα*), which reaching their expression peak at 3 h p.i, with lower levels observed at 24 h.p.i (Figs 2E, S1E). Moreover, we observed that DENV-2 infection in MDMs induced a significant increase in the mRNA expression of STAT1-dependent pro-inflammatory factors, including *IL7*, *IL15*, TNF-related apoptosis-inducing ligand (*TRAIL*), and B cell activating factor (*BAFF*) (Figs 2E, S1E). The mRNA expression of these genes reached their expression peak in the later stages of infection (24 h.p.i), with low levels observed during the eclipse period of infection, suggesting that induction of those inflammatory factors is dependent on active viral replication in MDMs.

Regarding CC/CXC-chemokines, DENV-infected MDMs exhibited distinct patterns. *CCL2* mRNA expression decreased at 5 h.p.i, followed by upregulation and reaching a peak at 24 h.p.i (Figs 2F, S1F). *CCL4* mRNA was highly induced during the first hours of infection (1.5 to 3 h.p.i), and peaked again at 24 h.p.i (Figs 2F, S1F). *CXCL1* and *CXCL8/IL8* mRNA levels reached their highest peak at 3 h.p.i, with the lowest expression levels observed at 10 h.p.i, remained low until 24 h.p.i (Figs 2F, S1F). Moreover, STAT1-dependent chemokines including *CCL5*, *CCL8, CXCL10, and CXCL11* showed a slight increase during the early stage of infection, the most significant increase occurred after 10 h.p.i, peaking at 24 h.p.i (Figs 2F, S1F). *CXCL9* was not expressed during the first 10 h.p.i, reaching its maximum expression peak at 24 h.p.i. Together, these results suggest that recognizing structural DENV-2-PAMPs during the eclipse period of DENV-2 infection promotes robust NF-kB-dependent pro-inflammatory factors expression. However, later in infection (24 h.p.i), the recognition of viral replication intermediates (ss/dsRNA) and/or structural DENV PAMPs may activate both NF-kB- and STAT1-dependent pathways, potentially playing a role in the induction of cytokine storm in DENV-2-infected MDMs.

### Sensing of DENV-2 RNAs promotes a dynamic, multi-type IFN-dependent antiviral response in human macrophages

**Fig 3:**
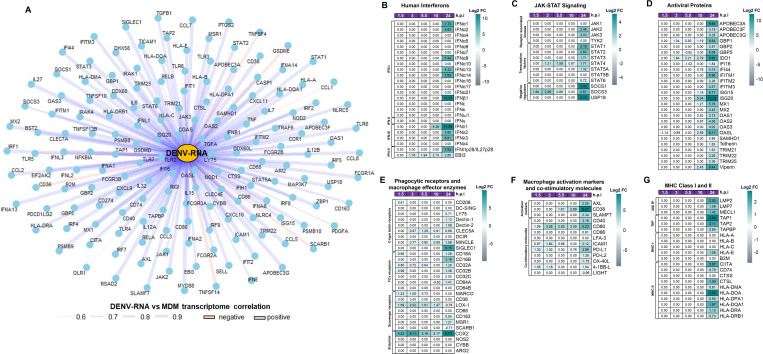
Active DENV-2 replication in human macrophages promotes a dynamic, multi-type IFN-dependent antiviral response. Human primary MDM cultures were either infected or not with DENV-2, as shown in Fig 1A. Gene expression (mRNA) is presented as Log2FC heatmaps. (A) correlation analysis between DENV RNA levels and transcriptomic changes in MDMs. **(B)** Interferons. **(C)** JAK-STAT signalling components. **(D)** ISGs encoding antiviral proteins. (E) phagocytic receptors and macrophage effector enzymes. (F) macrophage activation markers and co-stimulatory molecules. **(G)** MHC class I and II molecules. DEGs were identified using DESeq2 with a p-value < 0.05, and |Log2 Fold Change (DENV-2-infected MDMs/Control MDMs) | > 0.6.

As shown in Fig 1, the most prominent transcriptomic changes in DENV-2-infected MDMs coincided with the peak of viral RNA replication and induction of cytoplasmic viral RNA sensors at 24 h.p.i (Fig 2B), suggesting that the sensing of DENV-RNAs by PRRs may be a key driver of macrophage hyperactivation during infection. Supporting this hypothesis, we observed a strong and significant correlation between kinetics of DENV-2 RNA replication and induction of cellular mRNAs associated with the establishment of inflammatory and antiviral responses, including IFNs, antiviral proteins, costimulatory molecules, and other inflammatory mediators (Fig 3A). These findings indicated that PRR-mediated sensing of DENV-2 RNAs is critical for late-phase hyperactivation of innate inflammatory and antiviral pathways in infected MDMs.

Interferons are key inducers of the antiviral state in the cells, promoting the expression of STAT1-dependent pro-inflammatory factors that amplify innate and adaptive immune responses [50]. In line with the significant modulation of IRFs (Fig 2D), we found that DENV-2 infection induces high and significant mRNA expression of various type I IFNs, including *IFNα1*, *IFNα2*, *IFNα8*, *IFNα13*, *IFNα14*, and *IFNβ1*, as well as type III IFNs, including *IFN*λ*1*, *IFN*λ*2*, and *IFN*λ*3* in MDMs, but only at 24 h.p.i (Figs 3B, S2A). In contrast, both subunits of IL-27 (recently proposed by us as IFN-π, a type V IFN [50]), *IFNπp28/IL27p28* and *EBI3*, were induced increasingly from the first h.p.i, reaching their maximum expression peak at 24 h.p.i (Figs 3B, S2A). Type II IFN was not induced at any time evaluated. These results suggest active DENV-2 replication promotes a robust multi-type IFN-dependent antiviral response in human MDMs in late stages of infection.

In line with IFN expression, DENV-2 infection does not induce significant changes in the expression profile of JAK-STAT signaling components during the early stages of infection. However, at the later stages, there is an increased mRNA expression of *JAK2*, *JAK3*, *STAT1*, *STAT2*, and *STAT3*, with a maximum peak observed at 24 h.p.i (Figs 3C, S2B). In the same way, mRNA expression of JAK-STAT negative regulators, including *SOCS1*, *SOCS3*, and *USP18*, peaked at 24 h.p.i (Figs 3C, S2B). These results suggest active DENV-2 replication promotes robust IFN-dependent JAK-STAT signaling activation in MDMs. In agreement with those results, DENV-2 infection also significantly upregulated mRNA expression of genes encoding antiviral proteins (AVPs)/ISGs, including members of *APOBEC3*-, *GBP*-, *IFI*-, *IFITM*-, *MX*-, *OAS*-, and *TRIM*-family, as well as Indoleamine 2,3-dioxygenase 1 (*IDO1*), *ISG15*, *ISG20*, *Tetherin*, and *Viperin,* all of which peak at 24 h.p.i (Figs 3D, S2C). Altogether, results confirm that active DENV-2 replication promotes a robust multi-type IFN-dependent antiviral activity, and robust STAT1-dependent pro-inflammatory response in human MDMs (Fig 2).

### DENV-2 infection increases the phagocytic capacity of macrophages and promotes an adaptive immune response

The DENV-dependent cytokine storm is a complex immunopathological response in which different immune cells, including mononuclear phagocytes and T cells, play independent and complementary roles in promoting and amplifying inflammatory response in dengue patients [10,18,61]. Our results showed that DENV-2 infection induces a robust pro-inflammatory and antiviral response in human MDMs (Figs 2 and 3), promoting high expression of chemotactic factors and cytokines involved in T cell activation and differentiation (Fig 2E and 2F). These results suggest that DENV-2 infection induces macrophage activation to promote inflammatory response and antigen presentation to T cells. To explore this hypothesis, we evaluated the expression of macrophage markers associated with their activation profile and capability to promote antigen presentation to T cells.

Fig 3E notably revealed that DENV-2 infection significantly upregulated mRNA expression of genes encoding phagocytic receptors, including c-type lectin receptors such as Macrophage inducible Ca^2+^-dependent lectin receptor (*MINCLE*), Sialic acid binding Ig-like lectin 1 (*SIGLEC1*); FCγ receptors including *CD16A*/*FC*γ*R3A, CD32A/FC*γ*R2A, CD64A/FC*γ*R1A.* Furthermore, scavenger receptors such as *CD163* and Macrophage scavenger receptor 1 (*MSR1*), all of which peaked at 24 h.p.i (Figs 3E, S2D). Important, however, C-type lectin domain containing 5A (*CLEC5A*) mRNA expression peaks at 5.5 h.p.i, whereas *CD206* was significantly induced only at 1.5 h.p.i (Figs 3E, S2D). *DC-SIGN* was low expressed by MDMs, and its mRNA levels were not affected by DENV infection. Additionally, DENV-2 infection also upregulated mRNA expression of macrophage enzymes involved in the induction of inflammatory response, including Cyclooxygenase 2 (*COX2*), which was induced during the early hours of infection (1.5 to 3 h.p.i), followed by a decrease in expression levels between 5 and 10 h.p.i, and a subsequent peak at 24 h.p.i (Figs 3E, S2D). Together, results suggest that active DENV-2 replication induces an inflammatory activation profile in MDMs, increasing their phagocytic capability.

Furthermore, DENV-2 significantly upregulated mRNA expression of genes encoding cell-surface macrophage activation markers, including AXL receptor tyrosine kinase (*AXL*), *CD38*, SLAM family member 7 (*SLAMF7*), all of which peaked at 24 h.p.i (Figs 3F, S2E). Moreover, DENV-2-infected MDMs significantly upregulated the expression of costimulatory molecules such as *CD40*, *CD80*, *CD86*, and programmed death-ligand 1 (*PD-L1*) with a mRNA expression peaking at 24 h.p.i (Figs 3F, S2E). In contrast, *ICAM1* peaked at 3 h.p.i reaching its maximum expression at 24 h.p.i (Figs 3F, S2E). Furthermore, DENV-2 infection significantly increased the mRNA levels of 20S immunoproteasome (20S-IP) components, including Low molecular mass peptide 2 (*LMP2*), as well as Antigen peptide transporter (TAP) components including *TAP1.* These genes were exclusively transcribed at 24 h.p.i (Figs 3G, S2F). In contrast, non-significant changes were observed in the mRNA expression of *HLA-A* or Beta-2-microglobulin (*B2M*) in DENV-2-infected MDMs as compared to the control (Figs 3G, S2F). Notable, DENV-2 infection up-regulated mRNA expression of genes encoding MHC-II components, including class II major histocompatibility complex trans-activator (*CIITA*), *CD74*, *HLA-DPA1*, and *HLA-DRA* molecules, all of which peak at 24 h.p.i (Figs 3G, S2F). Together, results suggest that DENV-2-infected MDMs may contribute to antigens presentation to T cells, promoting an inflammatory macrophage activation profile and supporting the development of anti-DENV adaptive immune responses.

### Validation of RNA-seq data by RT-qPCT and ELISA

**Fig 4:**
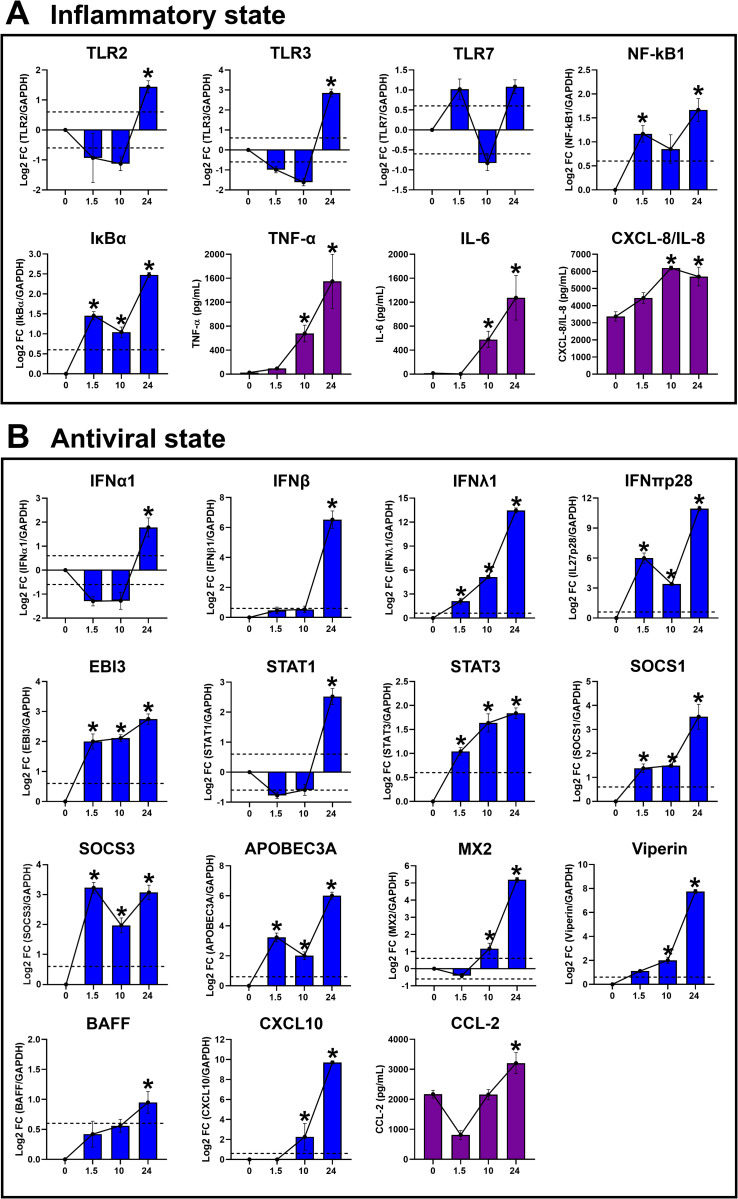
Experimental validation of RNA-seq results using RT-qPCR and ELISA. Human primary MDM cultures (n = 3) were either infected or left uninfected with DENV-2 (MOI = 5). Total RNA and culture supernatants were collected at 0 (control), 1.5, 10, and 24 h.p.i., and RT-qPCR and ELISA were performed. (A) mRNA levels of NF-κB-dependent inflammatory modulators and protein production. **(B)** IFN-STAT1-dependent antiviral and inflammatory factors. Data are presented as mean ± standard deviation (SD). Data normality was assessed using the Shapiro-Wilk test. Statistical analysis was performed using One-way ANOVA followed by Fisher’s least significant difference post-hoc test. Significant differences between control MDMs and DENV-2-infected MDMs were defined as p-value <0.05 (*).

To validate the RNA-Seq results, MDM cultures (n = 3) were infected with DENV-2 (MOI 5) for 1.5, 10, and 24 h.p.i, and uninfected MDMs (0) as control. The mRNA expression and protein production of selected DEG associated with inflammatory response identified by bulk RNA-Seq were measured using RT-qPCR or ELISA, respectively. The mRNA levels of *TLR2* and *TLR3* exhibited significantly increased expression at 24 h.p.i (Fig 4A), consistent with RNA-seq results (Fig 2). Similarly, mRNA expression of *TLR7*, *NF-kB1* and **IkB*α* displayed dynamic modulation, with initial peaks at 1.5 h.p.i, a decrease at 10 h.p.i, and subsequent peaks at 24 h.p.i (Fig 4A), aligning with RNA-seq results (Fig 2). However, protein levels of NF-kB-dependent pro-inflammatory factors, including TNF-α, IL-6, and CXCL-8/IL-8, were detected time-dependent, with all peaking at 24 h.p.i (Fig 4A). These findings diverged from RNA-Seq data, where mRNA expression of these factors was also evident at earlier time points (Fig 2). Together, these results suggest that the early recognition of DENV-PAMPs during the eclipse period of DENV-2 infection/replication promotes NF-kB-complex activation, driving the transcription of NF-kB-dependent pro-inflammatory factors (Fig 2E). However, based on our findings, we hypothesize that the translation and/or release of these inflammatory factors by infected cells predominantly occur later in the DENV-2 replicative cycle (Fig 4A).

Concerning the DEG involved in the induction of antiviral response, RT-qPCR analysis confirmed significant upregulation of *IFNα1*, *IFNβ1*, *IFN*λ*1*, *IFNπp28/IL27p28*, and *EBI3* mRNA levels, all peaking at 24 h.p.i in DENV-2-infected MDMs (Fig 4B), in agreement with RNA-seq results (Fig 3). Additionally, DENV-2 infection significantly increased transcriptional expression of genes encoding JAK-STAT signaling components, including *STAT1*, *STAT3*, *SOCS1*, and *SOCS3,* which also peaked at 24 h.p.i (Fig 4B). AVPs/ISGs such as *APOBEC3A*, *MX2*, and *Viperin*, along with STAT1-dependent pro-inflammatory factors like *BAFF* and *CXCL10*, reached maximum expression levels at 24 h.p.i (Fig 4B), corroborating bulk RNA-seq findings (Fig 3). Furthermore, the production of CCL-2 protein was validated in DENV-2-infected MDMs, with levels peaking at 24 h.p.i (Fig 4B). Altogether, these findings confirm the reliability of transcriptome data and demonstrate that DENV-2 infection promotes a dynamic NF-kB-dependent pro-inflammatory response, as well as a multi-type IFN-STAT1-dependent pro-inflammatory and antiviral states in human MDMs.

### DENV-2-infected Dendritic cells, and monocyte subsets from acute dengue patients promote innate antiviral response and cytokine storm

Monocytes (Mon) are the primary precursors of macrophages and dendritic cells in tissues [62]. Although Mon, macrophages, and dendritic cells exhibit distinct phenotypic and functional characteristics, those populations of mononuclear phagocytes are key targets for DENV infection and play a critical role in dengue immunopathogenesis and control [14,18,27,52,53]. These cells express a broad range of PRRs essential for sensing viral infections and initiating innate pro-inflammatory and antiviral responses (57)(62) [57,62]. To better characterize transcriptomic dysregulation and indirectly confirm the biological relevance of biomarkers identified in DENV-2-infected MDMs, we reanalysed the publicly available Microarray dataset (**GSE58278**) [52], performed in human Monocyte-derived dendritic cells (MDDCs) infected with DENV-2 NGC, at MOI 20 for 24 h.p.i. Also, we reanalysed the publicly available bulk RNA-seq dataset (**GSE176079**) [53], generated from sorted classical (CM, CD14^++^CD16^-^), intermediate (IM, CD14^++^CD16^+^), and non-classical (NM, CD14^Dim^CD16^+^) Mon subsets isolated from six acute dengue patients (ADP) and three healthy donors. This reanalysis aims to enhance the reliability of immunological activation state prediction in both dengue patients and *in vitro* models of DENV-infected macrophages and dendritic cells.

**Fig 5:**
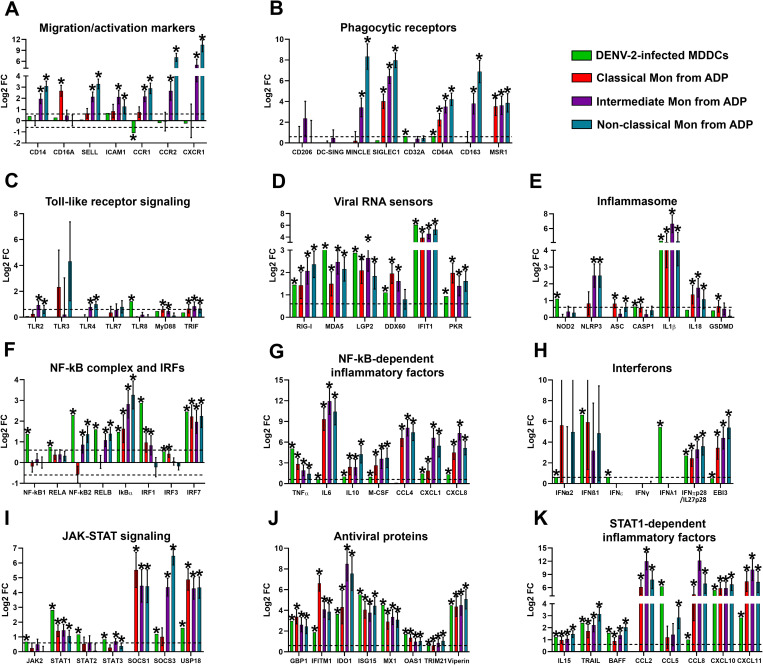
DENV-2-infected dendritic cells and monocyte subsets from acute dengue patients modulated PRR expression and promoted a robust NF-kB-dependent inflammatory response. We reanalysed publicly available Microarray data from human Monocyte-derived dendritic cells (MDDCs) infected with DENV-2 (MOI 20) for 24 hours. n = 3. In addition, we reanalysed a publicly available bulk RNA-seq dataset generated from sorted classical, intermediate, and non-classical Mon subsets isolated from six acute dengue patients (ADP) and three healthy donors. Gene expression (mRNA) is presented as Log2FC. The mRNA expression profiles included the following categories: **(A)** Monocyte activation markers. **(B)** Phagocytic receptors. **(C)** TLR signalling components. (D) viral RNA sensors. **(E)** Inflammasome components. **(F)** NF-kB complex and IRFs. **(G)** NF-kB-dependent inflammatory factors. **(H)** Interferons. **(I)** JAK-STAT signalling components. **(J)** ISGs encoding antiviral proteins. **(K)** STAT1-dependent inflammatory factors. DEGs were identified using GEO2R, with a p-value < 0.05 and |Log2 Fold Change (DENV-infected cells vs unstimulated cells) | > 0.6 (dotted line).

First, we evaluated the mRNA expression of activation markers in DENV-2-infected MDDCs and Mon subsets derived from ADP. As shown in Fig 5A, DENV-2-infected MDDCs only down-regulated *CCR1* mRNA expression. However, IM and NM subsets from ADP significantly up-regulated CD14 mRNA expression, whereas CM exhibited significant upregulation of *CD16A/FC*γ*R3A* mRNA expression compared to healthy donors. Further, IM and NM, but not CM subsets, significantly up-regulated mRNA expression of adhesion molecules, including L-selectin and *ICAM1,* as well as chemokine receptors involved in Mon migration from the blood into the tissues [63], including *CCR1*, *CCR2*, *CXCR1*, and *CXCR2* (Fig 5A). Additionally, all Mon subsets from ADP showed significant upregulation of mRNA levels for phagocytic receptors, also highly expressed by macrophages (Fig 5A), including *SIGLEC1*, *CD64A* and *MSR1* (Fig 5B). In contrast, *MINCLE* and *CD163* mRNA expression was selectively upregulated in IM and NM subsets, while *CD32A* expression remained unchanged (Fig 5B). However, Mon subsets from ADP did not modulate *CD206* and DCs activation marker *DC-SIGN* mRNA (Fig 5B). These results suggest that DENV infection may drive the migration of IM and NM subsets from the bloodstream into tissues, and their differentiation to macrophages during the acute phase of DENV infection in humans. This implies that those subsets of Mon could play pivotal roles in systemic DENV dissemination. Interestingly, DENV-2-infected MDDCs did not modulate the expression of c-type lectin receptors and scavenger receptors as Mon (Fig 5B) and MDMs (Fig 3A) but significantly upregulated FcγR, including *CD32A* and *CD64A*, suggesting that Mon subsets and MDMs induced a higher phagocytic activity during DENV infection as compared with MDDCs.

Consistent with RNA-seq results from DENV-2-infected MDMs, both IM and NM subsets from ADP exhibited significant upregulation of *TLR2* and *TLR4* mRNA levels (Fig 5C). However, non-significant changes were observed in *TLR7* and *TLR8* mRNA expression, whilst *TLR3* mRNA expression displayed non-significant yet considerable variability among patients. Notably, both CM and IM subsets exhibited upregulation of *MyD88* mRNA, whereas *TRIF* mRNA levels were significantly increased across all Mon subsets (Fig 5C). It is worth noting that DENV-2-infected MDDCs upregulated only *TLR8* mRNA. Moreover, in line with DENV-2-infected MDMs results (Fig 2B), the induction of viral RNA sensors involved in sensing DENV-RNAs, including *RIG-I*, *MDA5*, *LGP2*, *DDX60*, *IFIT1*, and *PKR* mRNA levels were upregulated by DENV-2-infected MDDCs and across all Mon subsets derived from ADP (Fig 5D). These results suggest that MDDCs and various Mon subsets established a robust antimicrobial state to counter DENV replication through upregulation of PRRs involved in sensing DENV-PAMPs, consistent with findings in DENV-2-infected MDMs (Fig 2).

Furthermore, transcriptomic analysis of DENV-2-infected MDDCs and Mon subsets from ADP revealed differential modulation of NLRP3-inflammasome components. While DENV-2-infected MDDCs significantly upregulated *NOD2* and *CASP1* mRNAs, IM and NM subsets significantly upregulated NLRP3 mRNA levels. Still, CM and NM subsets exhibited significant increases in ASC mRNA expression (Fig 5E). Notably, however, MDDCs and all Mon subsets displayed significant increases in *IL1β* and *IL18* mRNAs levels in response to DENV infection (Fig 5E). Only CM showed a slight increase in *GSDMD* mRNA expression (Fig 5E). These findings suggest a differential regulation of inflammasome components during DENV infection in MDDCs and Mon subsets. In addition, consistent with RNA-seq data from DENV-2-infected MDMs, DENV-2-infected MDDCs and IM and NM subsets from ADP showed significant increases in mRNAs expression of NF-kB-complex and IRFs components, including *NF-kB2* and *RELB*, whilst **IkB*α* and *IRF7* mRNA expression was significantly upregulated across all Mon subsets and MDDCs (Fig 5F). While MDDCs, CM and IM subsets demonstrated significant increases in *IRF1* mRNA levels in response to DENV infection, *NF-kB1*, *RELA*, and *IRF3* mRNA levels were significantly induced in DENV-2-infected MDDCs but remained unaltered across Mon subsets (Fig 5F). Moreover, DENV-2-infected MDDCs and all Mon subsets from ADP exhibited significant upregulation of NF-kB-dependent inflammatory factors, including cytokines such as *TNFα*, *IL6*, *IL10*, and *M-CSF*, as well as chemokines including *CCL4*, *CXCL1*, and *CXCL8/IL8* (Fig 5G), as was observed in DENV-2-infected MDMs (Fig 2). These results suggest that all population of mononuclear phagocytes, including Mon subsets, dendritic cells, and macrophages play a key role in sensing DENV infection to promote robust and systemic NF-kB-dependent inflammatory responses.

Notably, DENV-2-infected MDDCs significantly upregulated the expression of different types of IFNs, including IFN-I (*IFNα2*, *IFNβ1*, and *IFN*ε**), IFN-III (**IFNλ1**), and both subunits of IFN-V (*IFNπp28/IL27p28* and *EBI3*) (Fig 5H), suggesting that like DENV-2-infected MDMs (Fig 4), MDDCs induce a multi-type IFN-dependent antiviral response to DENV infection. However, Mon subsets from ADP exhibited considerable variability and non-significant changes in *IFNα2* and *IFNβ1* mRNA levels, without induction of IFN-III (Fig 5H). Important however, all Mon subsets from ADP showed significant expression of IFN-π/IL-27 subunits (*IFNπp28/IL27p28* and *EBI3*) (Fig 5H). In line with those results, DENV-2-infected MDDCs upregulated the expression of JAK-STAT signaling components involved in IFN-I/IFN-III/IFN-V signaling transduction, including *JAK2*, *STAT1*, *STAT2*, *STAT3*, *SOCS3* and *USP18* (Fig 5I). However, all Mon subsets from ADP only upregulated mRNA expression of *STAT1*, *SOCS1*, and USP18 (Fig 5I). In contrast, *STAT3* and *SOCS3* mRNA expression was induced only in IM and NM subsets (Fig 5I). In agreement with IFNs expression, DENV-2-infected MDDCs and all Mon subsets from ADP upregulated mRNA levels of antiviral ISGs/AVPs, including *GBP1*, *IFITM1*, *IDO1*, *ISG15*, *MX1*, *OAS1*, *TRIM22*, and *Viperin* (Fig 5J), indicating the induction of a robust antiviral state. Additionally, DENV-2-infected MDDCs and all Mon subsets significantly upregulated STAT1-dependent inflammatory factors, including cytokines such as *IL15*, *TRAIL*, and *BAFF,* along with chemokines *CCL8*, *CXCL10,* and *CXCL11* (Fig 5K). However, *CCL2* mRNA was upregulated only by Mon subset, whereas *CCL5* mRNA was induced by DENV-2-infected MDDCs and NM subset from ADP (Fig 5K). These results confirm that DENV infection in both MDDCs and Mon subsets activate IFN-dependent JAK-STAT signaling and induce robust STAT1-dependent pro-inflammatory and antiviral responses, as was observed in DENV-2-infected MDMs (Fig 3)

**Fig 6:**
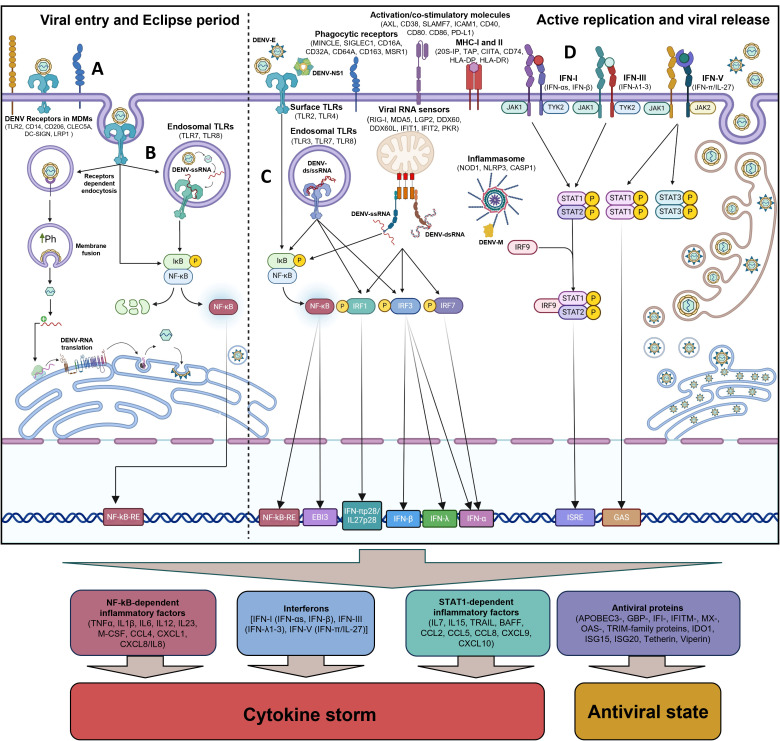
DENV-2 infection triggers a robust innate immune response in human MDMs via multiple PRRs, NF-κB, and IFN-STAT1 signaling pathways. **(A)** Human MDMs express receptors that facilitate DENV entry. **(B)** Early during infection, TLR2 and TLR7 recognized DENV-RNA and other PAMPs, initiating NF-κB-dependent inflammatory responses. **(C)** Following the eclipse phase, active viral replication leads to immune hyperactivation, characterized by upregulation of multiple PRRs, including TLRs, NLRs, and viral RNA sensors, further amplifying inflammation. **(D)** DENV-2-infected MDMs activate multiple IFN pathways, producing type I/III and V IFNs, which trigger STAT1-mediated activating response, including ISGs and STAT1-dependent inflammatory factors.

Together, these results showed that *in vitro* DENV-2-infected MDDCs and all Mon subsets from ADP play a pivotal role in promoting both NF-kB- and IFN-STAT1-dependent inflammatory responses. This aligns with findings in DENV-2-infected MDMs (Figs 3 and 4), confirming the key role of all mononuclear phagocytes in promoting cytokine storm and immunopathogenesis during DENV infection in humans, both *in vitro* and *in vivo*. The results are summarized in Fig 6.

## Discussion

Dengue pathogenesis is driven mainly by immunopathological mechanisms, primarily mediated by exacerbated and uncontrolled inflammatory responses induced by mononuclear phagocytes, such as monocytes, macrophages, and dendritic cells [15,17,18,27,55]. This study uses bulk RNA-seq and Microarray data to present comprehensive immune profiles from *in vitro* DENV-2-infected MDMs and MDDCs, and monocyte subsets derived from acute dengue patients. Specifically, we describe the early and late events associated with DENV-2-induced cytokine storm in human macrophages. Interestingly, we found that human MDMs expressed different DENV receptors involved in viral entry into the cells, including CD206, DC-SIGN, CLEC5A, LRP1, TLR2, and CD14 (Fig 6A). Additionally, during the eclipse period of the DENV-2 replication cycle in human MDMs, defined as the period between viral entry into the target cell and the beginning of active viral RNA replication and release of newly produced virions (3 to 5.5 h.p.i), we observed a decreased in the accumulation of DENV-2 RNA copies within MDMs. Additionally, we identified a significant increase in the TLR2 and TLR7 mRNA levels, along with activation of NF-kB-complex during the eclipse period of infection, suggesting an essential role of those TLRs in sensing DENV-ssRNA and/or structural PAMPs in viral particles by macrophages (Fig 6B). However, whilst TLR2 expression significantly increased in IM and NM subsets from acute dengue patients, TLR7 was not (Fig 5). In line with those results, Aguilar-Briseño et al. [18,54] reported that TLR2 plays a key role in sensing DENV-E protein, promoting viral entry into monocytes, and triggering a robust NF-kB-dependent inflammatory response. Moreover, early sensing of DENV-ssRNA by TLR7 and/or TLR8 could play a synergistic effect with TLR2 to promote NF-kB-dependent inflammatory response and endothelial cell activation by DENV-2-infected monocytes/PBMCs (submitted manuscript). Moreover, a significant increase in TLR2 expression has been documented in a DENV-infected paediatric cohort and was identified as a prognostic value of disease pathogenesis [18]. Furthermore, after the eclipse period of DENV-2 replication in MDMs, we observed that the active DENV-RNA replication and release of viral particles (from 10 to 24 h.p.i) promote macrophage hyperactivation, leading to high expression of multiple PRRs, followed by a secondary wave in the inflammatory response. Among these PRRs, Toll-like receptors, including *TLR2*, *TLR3*, *TLR4*, *TLR7*, and *TLR8*; NLRs such as *NOD2* and *NLRP3*; and viral RNA sensors such as *RIG-I*, *MDA5*, *DDX60*, *IFIT1*, and *PKR* were highly induced by infected MDMs (Fig 6C). All these PRRs have been shown to play a role in sensing different structural DENV PAMPs and/or intermediate viral replication (ss/dsRNAs) in infected cells [18,33–39]. Moreover, consistent with previous reports [64], we observed a similar pattern in the induction of PRRs and phagocytic receptors in Mon subsets derived from acute dengue patients and in DENV-2-infected MDMs at 24 h.p.i. This suggests that these PRRs and phagocytic receptors play a critical role in sensing/capturing DENV particles by MDMs and Mon, thereby promoting innate pro-inflammatory and antiviral responses *in vitro* and *in vivo*. Noteworthy, however, although DENV-2-infected MDDCs induce a high expression of viral RNA sensors, they did not induce significant changes in the expression of *TLR2*, *TLR3*, *TLR4*, *TLR7*, and phagocytosis receptors as observed in monocytes and MDMs, suggesting that DENV infection promotes a lower antimicrobial activation in dendritic cells, as compared to monocytes and macrophages.

NF-kB-dependent inflammatory factors have been canonically considered as the key component of DENV-dependent cytokine storm since TNF-α and IL-1β produced by DENV-infected cells promote endothelial cells hyperactivation and disruption of endothelium integrity [9,10,12,18,65,66]. Moreover, high serum levels of TNF-α, IL-1β, IL-6, CXCL-8/IL-8, and IL-10 correlated with severity of the disease in dengue patients [8,9,11,67], showing a preponderant role of NF-kB-dependent inflammatory response in dengue immunopathogenesis. In line with those reports, we observed that both DENV-infected MDMs and MDDCs, as well as Mon subsets from acute dengue patients, exhibit high expression of the NF-kB complex and its target genes. These include cytokines such as *TNFα*, *IL1β*, *IL6*, *IL10*, and *M-CSF,* as well as chemokines like *CCL4*, *CXCL1*, and *CXCL8/IL8* (Fig 6B and 6C). This confirms the induction of robust NF-kB-dependent inflammatory responses by all populations of mononuclear phagocytes in response to DENV infection, both *in vitro* and *in vivo*. In agreement with these findings, our research group previously reported that DENV-2 infection in human MDMs and MDDCs promotes high protein production of NF-kB-dependent inflammatory cytokines, including TNF-α, IL-1β, IL-6, CXCL-8/IL-8, and IL-10 [26,27,38]. Moreover, several studies have shown that DENV infection in MDMs, MDDCs, and Mon induces a broad range of NF-kB-dependent pro-inflammatory factors [18,27,41,52–54]. Additionally, Mohamad Al Kadi et al. [68], using single-cell sequencing of DENV (DV3P12/08)-infected FN-α/β/γ receptor knockout (IFN-α/β/γRKO) mice, which lack both type I and II IFN signalling, demonstrated that various immune cell populations contribute to NF-kB-driven inflammatory responses. These include M2-like macrophages, DCs, and neutrophils, which promote Il-1β production, whereas M1-like macrophages primarily produce Tnf-α, Il-6, and Il-23, contributing to the cytokine storm. However, this mouse model lacks IFN-STAT1-dependent antiviral and inflammatory responses, which are critical components of cytokine storm induced by mononuclear phagocytes in humans. These observations support our results and highlight the critical role of mononuclear phagocytes in driving local and systemic NF-kB-dependent inflammatory responses to DENV infection. Notably, the activation of NF-kB complex during the eclipse phase of DENV-2 replication in MDMs results in high transcription of NF-kB-dependent cytokines and chemokines involved in the establishment of inflammatory response. However, the secretion of TNF-α, IL-6, and CXCL-8/IL-8 by DENV-2-infected MDMs occurred at late stages of infection, suggesting that the translation and/or secretion of these inflammatory factors are delayed in DENV-2 replicative cycle. This observation implies that early activation of NF-kB induced by DENV-2 infection may serve as a critical priming signal to initiate a functional inflammatory response, ultimately contributing to the cytokine storm observed in the late stage of DENV-2 infection in MDMs.

In parallel with NF-kB-dependent inflammatory response, we demonstrate that both DENV-2-infected MDMs and MDDCs, and Mon subsets from ADP promote robust IFN-STAT1-dependent antiviral and inflammatory responses (Fig 6D). Important however, the nature of these responses was cell-type specific since DENV-2-infected MDMs and MDDCs exhibited high expression of various type I IFNs (IFN-αs, IFN-β, and/or IFN-ε), IFN-III (IFN-λ1-3), and IFN-V (IFN-π/IL-27) (Fig 6D). In contrast, Mon subsets from ADP only upregulated both IFN-π/IL-27 subunits (IFN-πp28/IL27p28 and EBI3), suggesting a more potent induction of multi-IFN-depend antiviral response to DENV infection by MDMs and MDDCs, as compared to Mon subsets from ADP. In line with this result, various authors have shown that DENV infection promotes IFN-α, IFN-β and/or IFN-λ expression by human MDMs and MDDCs in response to DENV infection [41,69]. However, *in vitro* studies of DENV infection in Mon/PBMCs demonstrated upregulation of IFN-I and/or IFN-III production [18], unlike Mon subsets from ADP. This suggests a differential response of Mon to DENV infection *in vitro* versus *in vivo.* Moreover, we provide the first report of IFN-π/IL-27 as an inductor of antiviral response induced by mononuclear phagocytes in response to DENV infection. Previous reports have demonstrated the antiviral properties of IFN-π/IL-27 and their capacity to establish an antiviral state and control the replication of DENV-2 and Zika virus in MDMs and Mon [50,61]. These findings confirm that the induction of IFN-π/IL-27 by Mon, MDMs and MDDCs during the acute phase of DENV infection in humans may be crucial in promoting local and systemic antiviral response to control viral replication.

Interferons have been shown to play a key role in controlling DENV replication into host cells by induction of different ISGs encoding AVPs, including OAS2, ISG15, and Viperin, which have been shown to play a role in controlling DENV replication in different steps of the viral cycle [70–72]. Interestingly, both DENV-2-infected MDMs and MDDCs, and Mon subsets from ADP activate the JAK-STAT signaling pathway, resulting in the high expression of various AVPs (Fig 6D). This activation establishes a robust antiviral state, enabling mononuclear phagocyte populations to control DENV replication, both *in vitro* and *in vivo*. In agreement with our findings, previous reports have demonstrated that stimulation of various cell populations with different types of IFNs induces the expression of ISGs/AVPs, thereby contributing to the control of DENV infection [35,37–39,50]. Notably, the early recognition of DENV-associated PAMPs (such as viral proteins, ssRNA) by TLR2, TLR7/8, and or RIG-I during the entry-eclipse phase of DENV-2 infection in MDMs appears to induce only NF-KB-dependent inflammatory response, without triggering IFN production or the ISGs/AVPs expression. This limited response occurs despite the strong and significant induction of SOCS3, a key negative regulator of inflammation, during the eclipse phase, suggesting a potential suppression of IFN production and/or JAK-STAT signaling during the early stages of infection. This may allow the virus to evade the innate antiviral response during the initial period of infection. However, once DENV-2 fully colonizes the MDMs, the virus initiates a burst of replication that drives rapid macrophage hyperactivation through engagement of multiple PRRs, resulting in the induction of both NF-KB and IFN-STAT1-dependent immune pathways. In line with that hypothesis, several studies have reported that specific DENV-NS proteins interfere with RIG-I-mediated IFN-I production and JAK-STAT signalling transduction in infected cells [73,74].

In line with the induction of an antiviral state, activation of JAK-STAT signaling by both DENV-2-infected MDMs and MDDCs, and Mon subsets from ADP, also promotes a high expression of STAT1-dependent inflammatory factors (Fig 6D). These include *IL15*, which has been involved in enhancing NK cell activation, proliferation, and their capacity to kill virus-infected cells [75,76]; *TRAIL*, which promotes inflammatory response and induces apoptosis in tumoral cells [77]; and *BAFF*, which facilitates B cell activation, proliferation, and antibodies production [78,79]. Moreover, both MDMs, MDDCs, and Mon subsets induced high expression of STAT1-dependent chemokines, including *CCL2*, *CCL5*, *CCL8*, *CXCL10,* and/or *CXCL11* (Fig 6D) which play a key role in the recruitment of different immune cell populations at the site of infections, including Mon, NK cells, T cells, and B cells, leading to the amplification of innate and adaptive immune responses [80,81]. Moreover, serum levels of IL-15, CCL-2, CXCL-10, and CXCL-11 correlated with dengue severity in humans [8,9]. Therefore, although IFN-STAT1-dependent pro-inflammatory and antiviral factors could play a key role in promoting more complex and effective immune response to control DENV infection, an exacerbated or uncontrolled production of IFNs and STAT1-dependent inflammatory factors may promote excessive inflammation and immunopathology, as has been observed in other viral infections, including those induced by chikungunya virus (CHIKV), and Severe acute respiratory syndrome coronavirus 2 SARS-CoV-2 [44,63]. Moreover, Hong et al. [82], using single-cell RNA sequencing of endothelial cells from a murine model of LPS-induced acute lung injury (Ppard^EC-KO^ mice), reported that elevated STAT1-dependent CXCL-10 production was associated with increased endothelial permeability, promoting pulmonary vascular leakage. Moreover, the authors showed that administration of either a neutralizing anti-CXCL-10 antibody or the CXCL-10 receptor antagonist AMG487 suppressed both LPS-induced lung inflammation and vascular leakage in Ppard^EC-KO^-mice. These finding confirm an immunopathological role for CXCL-10 in LPS-induced acute lung injury by promoting endothelial permeability and vascular leakage *in vivo*.

In consequence, the overproduction of different types of IFNs and STAT1-dependent inflammatory factors by monocytes, macrophages, and/or dendritic cells in response to DENV infection also contributes to the induction of pathological immune response, which is complementary to NF-kB-dependent inflammation and leads to an entire cytokine storm, both *in vitro* and *in vivo*. Furthermore, in line with our hypothesis, numerous studies have demonstrated that monocytes, macrophages, and DCs play a pivotal role in triggering cytokine storms during severe viral infections by hyperactivating the immune response. For instance, in Ebola virus infection, these cells recognize viral PAMPs through TLRs, RLRs, and other pattern recognition receptors, leading to excessive pro-inflammatory cytokine release, endothelial hyperactivation, and hemorrhage [83,84]. Similarly, in severe Influenza A infections, alveolar macrophages, DCs, and circulating monocytes drive an overproduction of inflammatory cytokines, exacerbating lung injury and inflammation [85,86]. During SARS-CoV-2 infection, monocytes and macrophages detect viral particles and produce IL-6, IL-1β, and IFN-π/IL-27 through TLR, NF-κB activation, and inflammasome assembly, contributing to acute respiratory distress syndrome (ARDS) and severe COVID-19 [63,87,88]. Yellow fever virus infection also triggers massive cytokine secretion through activation of hepatic macrophages, promoting systemic inflammation and multi-organ failure [89–91]. Collectively, these findings highlight mononuclear phagocytes as promising targets for future immunotherapies aimed at controlling pathological NF-kB- and STAT1-dependent immune responses and mitigating hyperinflammation in severe viral infections.

### Limitations of the study

This study has several limitations:

- Although we used the highly pathogenic DENV-2 NGC strain as a model of severe DENV infection in MDMs, it remains to be determined whether the immune response elicited by MDMs in response to this strain is comparable to that induced by other DENV serotype or by clinical isolates from dengue patients, which may better reflect the viral fitness and immune evasion strategies of currently circulating strains.
- Most severe DENV-2 infections in humans occur during secondary infections, in which antibody-dependent enhancement (ADE) mediated by non-neutralizing heterotypic anti-DENV antibodies plays a central role in pathogenesis by promoting hyper-infection of mononuclear phagocytes through FCγ receptors (CD32, CD16). However, this study did not assess the impact of ADE on cytokine storm induction in DENV-2-infected MDMs. Although mononuclear phagocytes are key contributors to dengue immunopathogenesis, other immune cells, such as B cells, T cells, and NK cells, may also actively participate in the development of the cytokine storm. Further investigation is needed to evaluate the role of these immune subsets in the broader inflammatory response to DENV infection.

## Conclusions

Altogether, results suggest that recognizing viral PAMPs during the eclipse period of DENV-2 infection promotes a robust NF-kB-dependent pro-inflammatory response in MDMs. However, at later stages of infection, the hyperactivation of PRRs involved in recognizing structural DENV-PAMPs and/or viral replication intermediates induces both NF-kB- and IFN-STAT1-dependent pro-inflammatory responses, leading to a cytokine storm. Similar results were observed on *in vitro* DENV-2-infected MDDCs and Mon subsets from acute dengue patients. Therefore, our results demonstrate that the overactivation of multiple PRRs in monocytes, macrophages, and dendritic cells during the acute phase of DENV infection plays a critical role in promoting local and systemic NF-kB- and IFN-STAT1-dependent inflammatory responses, leading to cytokine storm and immunopathology, both *in vitro* and *in vivo*. This suggests that these populations of mononuclear phagocytes could be potential targets for future immunotherapies to modulate the inflammatory response to DENV infection.

## Supporting information

S1 Fig Human primary MDM cultures were either infected or not with DENV-2, as shown in Fig 1A. Gene expression (mRNA) is presented as transcripts per million (TPM). mRNA expression levels are shown for: (A) TLR signaling components. (B) cytoplasmic viral RNA sensors. (C) NOD-like receptors and inflammasome components. (D) NF-kB complex and IRFs. (E) cytokines and growth factors. (F) chemokines. TPM values are presented as mean ± SD. DEGs were identified using DESeq2 with a p-value < 0.05, and |Log2 Fold Change (DENV-2-infected MDMs vs Control MDMs)| > 0.6, and are indicated as *.
(JPG)

S2 Fig Human primary MDM cultures were either infected or not with DENV-2, as shown in Fig 1A. Gene expression (mRNA) is presented as transcripts per million (TPM). (A) mRNA expression of Interferons. (B) JAK-STAT signaling components. (C) ISGs encoding antiviral proteins. (D) phagocytic receptors and macrophage effector enzymes. (E) macrophage activation markers and co-stimulatory molecules. (F) MHC class I and II molecules. TPM values are presented as mean ± SD. DEGs were identified using DESeq2 with a p-value < 0.05, and |Log2 Fold Change (DENV-2-infected MDMs vs Control MDMs)| > 0.6, and are indicated with an *.
(JPG)

S1 Table Information on primer sequences for the qPCR assay in this study. (XLSX)

## References

[1] Robert MA, Stewart-Ibarra AM, Estallo EL. Climate change and viral emergence: evidence from Aedes-borne arboviruses. Curr Opin Virol. 2020;40:41–7. doi: 10.1016/j.coviro.2020.05.001 32569752 PMC7305058

[2] Whitehorn J, Yacoub S. Global warming and arboviral infections. Clin Med (Lond). 2019;19(2):149–52. doi: 10.7861/clinmedicine.19-2-149 30872300 PMC6454362

[3] Ramos-Castañeda J, Barreto Dos Santos F, Martínez-Vega R, Galvão de Araujo JM, Joint G, Sarti E. Dengue in Latin America: Systematic Review of Molecular Epidemiological Trends. PLoS Negl Trop Dis. 2017;11(1):e0005224. doi: 10.1371/journal.pntd.0005224 28068335 PMC5221820

[4] Thomas SJ, Strickman D, Vaughn DW. Dengue epidemiology: virus epidemiology, ecology, and emergence. Adv Virus Res. 2003;61:235–89. 14714434 10.1016/s0065-3527(03)61006-7

[5] Nakase T, Giovanetti M, Obolski U, Lourenço J. Population at risk of dengue virus transmission has increased due to coupled climate factors and population growth. Commun Earth Environ. 2024;5(1). doi: 10.1038/s43247-024-01639-6

[6] Situación epidemiológica del dengue - OPS/OMS | Organización Panamericana de la Salud [Internet]. Available from: https://www.paho.org/es/arbo-portal/dengue/situacion-epidemiologica-dengue

[7] Organization PAH. Dengue: guidelines for patient care in the Region of the Americas. 2nd ed. Vol. 8, Disaster Prevention and Management: An International Journal. 2016. p. 996–1000.

[8] Srikiatkhachorn A, Green S. Markers of dengue disease severity. Curr Top Microbiol Immunol. 2010;338:67–82. doi: 10.1007/978-3-642-02215-9_6 19802579

[9] Puc I, Ho TC, Yen KL, Vats A, Tsai JJ, Chen PL, et al. Cytokine signature of dengue patients at different severity of the disease. Int J Mol Sci. 2021;22(6):1–15. 10.3390/ijms22062879 PMC7999441 33809042

[10] Kurane I. Dengue hemorrhagic fever with special emphasis on immunopathogenesis. Comp Immunol Microbiol Infect Dis. 2007;30(5–6):329–40. doi: 10.1016/j.cimid.2007.05.010 17645944

[11] Malavige GN, Huang L-C, Salimi M, Gomes L, Jayaratne SD, Ogg GS. Cellular and cytokine correlates of severe dengue infection. PLoS One. 2012;7(11):e50387. doi: 10.1371/journal.pone.0050387 23209731 PMC3510251

[12] Butthep P, Chunhakan S, Yoksan S, Tangnararatchakit K, Chuansumrit A. Alteration of cytokines and chemokines during febrile episodes associated with endothelial cell damage and plasma leakage in dengue hemorrhagic fever. Pediatr Infect Dis J. 2012;31(12):232–8. 10.1097/INF.0b013e31826fd456 22926216

[13] Halstead SB. Delivering safe dengue vaccines. Lancet Glob Health. 2024;12(8):e1229–30. doi: 10.1016/S2214-109X(24)00226-2 39030056

[14] Miller JL, de Wet BJM, Martinez-Pomares L, Radcliffe CM, Dwek RA, Rudd PM, et al. The mannose receptor mediates dengue virus infection of macrophages. PLoS Pathog. 2008;4(2):e17. doi: 10.1371/journal.ppat.0040017 18266465 PMC2233670

[15] Lozach P-Y, Burleigh L, Staropoli I, Navarro-Sanchez E, Harriague J, Virelizier J-L, et al. Dendritic cell-specific intercellular adhesion molecule 3-grabbing non-integrin (DC-SIGN)-mediated enhancement of dengue virus infection is independent of DC-SIGN internalization signals. J Biol Chem. 2005;280(25):23698–708. doi: 10.1074/jbc.M504337200 15855154

[16] Huerta V, Martin AM, Sarría M, Guirola O, Yero A, Ramos Y, et al. The Low-Density Lipoprotein Receptor-Related Protein-1 Is Essential for Dengue Virus Infection. Viruses. 2024;16(11):1692. doi: 10.3390/v16111692 39599807 PMC11599027

[17] Lo Y-L, Liou G-G, Lyu J-H, Hsiao M, Hsu T-L, Wong C-H. Dengue Virus Infection Is through a Cooperative Interaction between a Mannose Receptor and CLEC5A on Macrophage as a Multivalent Hetero-Complex. PLoS One. 2016;11(11):e0166474. doi: 10.1371/journal.pone.0166474 27832191 PMC5104462

[18] Aguilar-Briseño JA, Upasani V, Ellen BMT, Moser J, Pauzuolis M, Ruiz-Silva M, et al. TLR2 on blood monocytes senses dengue virus infection and its expression correlates with disease pathogenesis. Nat Commun. 2020;11(1):3177. doi: 10.1038/s41467-020-16849-7 32576819 PMC7311456

[19] Piccini LE, Castilla V, Damonte EB. Dengue-3 Virus Entry into Vero Cells: Role of Clathrin-Mediated Endocytosis in the Outcome of Infection. PLoS One. 2015;10(10):e0140824. doi: 10.1371/journal.pone.0140824 26469784 PMC4607419

[20] Christian EA, Kahle KM, Mattia K, Puffer BA, Pfaff JM, Miller A, et al. Atomic-level functional model of dengue virus Envelope protein infectivity. Proc Natl Acad Sci U S A. 2013;110(46):18662–7. doi: 10.1073/pnas.1310962110 24158478 PMC3831943

[21] Osawa T, Aoki M, Ehara H, Sekine S-I. Structures of dengue virus RNA replicase complexes. Mol Cell. 2023;83(15):2781-2791.e4. doi: 10.1016/j.molcel.2023.06.023 37478848

[22] van den Elsen K, Quek JP, Luo D. Molecular Insights into the Flavivirus Replication Complex. Viruses. 2021;13(6):956. doi: 10.3390/v13060956 34064113 PMC8224304

[23] Chiu W-W, Kinney RM, Dreher TW. Control of translation by the 5’- and 3’-terminal regions of the dengue virus genome. J Virol. 2005;79(13):8303–15. doi: 10.1128/JVI.79.13.8303-8315.2005 15956576 PMC1143759

[24] Renner M, Dejnirattisai W, Carrique L, Martin IS, Karia D, Ilca SL, et al. Flavivirus maturation leads to the formation of an occupied lipid pocket in the surface glycoproteins. Nat Commun. 2021;12(1):1238. doi: 10.1038/s41467-021-21505-9 33623019 PMC7902656

[25] Zicari S, Arakelyan A, Fitzgerald W, Zaitseva E, Chernomordik LV, Margolis L, et al. Evaluation of the maturation of individual Dengue virions with flow virometry. Virology. 2016;488:20–7. doi: 10.1016/j.virol.2015.10.021 26590794 PMC4744565

[26] Giraldo DM, Cardona A, Urcuqui-Inchima S. High-dose of vitamin D supplement is associated with reduced susceptibility of monocyte-derived macrophages to dengue virus infection and pro-inflammatory cytokine production: An exploratory study. Clin Chim Acta. 2018;478:140–51. doi: 10.1016/j.cca.2017.12.044 29289621

[27] Martínez-Moreno J, Hernandez JC, Urcuqui-Inchima S. Effect of high doses of vitamin D supplementation on dengue virus replication, Toll-like receptor expression, and cytokine profiles on dendritic cells. Mol Cell Biochem. 2020;464(1–2):169–80. doi: 10.1007/s11010-019-03658-w 31758375

[28] Tsai Y-T, Chang S-Y, Lee C-N, Kao C-L. Human TLR3 recognizes dengue virus and modulates viral replication in vitro. Cell Microbiol. 2009;11(4):604–15. doi: 10.1111/j.1462-5822.2008.01277.x 19134117

[29] Modhiran N, Watterson D, Muller DA, Panetta AK, Sester DP, Liu L, et al. Dengue virus NS1 protein activates cells via Toll-like receptor 4 and disrupts endothelial cell monolayer integrity. Sci Transl Med. 2015;7(304):304ra142. doi: 10.1126/scitranslmed.aaa3863 26355031

[30] Posadas-Mondragón A, Aguilar-Faisal JL, Zuñiga G, Magaña JJ, Santiago-Cruz JA, Guillén-Salomón E, et al. Association of Genetic Polymorphisms in TLR3, TLR4, TLR7, and TLR8 with the Clinical Forms of Dengue in Patients from Veracruz, Mexico. Viruses. 2020;12(11):1230. doi: 10.3390/v12111230 33138336 PMC7694044

[31] Wang JP, Liu P, Latz E, Golenbock DT, Finberg RW, Libraty DH. Flavivirus activation of plasmacytoid dendritic cells delineates key elements of TLR7 signaling beyond endosomal recognition. J Immunol. 2006;177(10):7114–21. 17082628 10.4049/jimmunol.177.10.7114

[32] Domínguez-Martínez DA, Pérez-Flores MS, Núñez-Avellaneda D, Torres-Flores JM, León-Avila G, García-Pérez BE, et al. NOD2 Responds to Dengue Virus Type 2 Infection in Macrophage-like Cells Interacting with MAVS Adaptor and Affecting IFN-α Production and Virus Titers. Pathogens. 2024;13(4):306. doi: 10.3390/pathogens13040306 38668261 PMC11054756

[33] Pan P, Zhang Q, Liu W, Wang W, Lao Z, Zhang W, et al. Dengue Virus M Protein Promotes NLRP3 Inflammasome Activation To Induce Vascular Leakage in Mice. J Virol. 2019;93(21):e00996-19. doi: 10.1128/JVI.00996-19 31413130 PMC6803285

[34] Chazal M, Beauclair G, Gracias S, Najburg V, Simon-Lorière E, Tangy F, et al. RIG-I Recognizes the 5’ Region of Dengue and Zika Virus Genomes. Cell Rep. 2018;24(2):320–8. doi: 10.1016/j.celrep.2018.06.047 29996094

[35] Zhang J, Sze DM-Y, Yung BY-M, Tang P, Chen W-J, Chan K-H, et al. Distinct expression of interferon-induced protein with tetratricopeptide repeats (IFIT) 1/2/3 and other antiviral genes between subsets of dendritic cells induced by dengue virus 2 infection. Immunology. 2016;148(4):363–76. doi: 10.1111/imm.12615 27135915 PMC4948034

[36] Ricciardi-Jorge T, da Rocha EL, Gonzalez-Kozlova E, Rodrigues-Luiz GF, Ferguson BJ, Sweeney T, et al. PKR-mediated stress response enhances dengue and Zika virus replication. mBio. 2023;14(5):e0093423. doi: 10.1128/mbio.00934-23 37732809 PMC10653888

[37] Olagnier D, Scholte FEM, Chiang C, Albulescu IC, Nichols C, He Z, et al. Inhibition of dengue and chikungunya virus infections by RIG-I-mediated type I interferon-independent stimulation of the innate antiviral response. J Virol. 2014;88(8):4180–94. 24478443 10.1128/JVI.03114-13 PMC3993760

[38] Castillo JA, Urcuqui-Inchima S. Vitamin D modulates inflammatory response of DENV-2-infected macrophages by inhibiting the expression of inflammatory-liked miRNAs. Pathog Glob Health. 2023;117(2):167–80. doi: 10.1080/20477724.2022.2101840 35850625 PMC9970239

[39] Palma-Ocampo HK, Flores-Alonso JC, Vallejo-Ruiz V, Reyes-Leyva J, Flores-Mendoza L, Herrera-Camacho I, et al. Interferon lambda inhibits dengue virus replication in epithelial cells. Virol J [Internet]. 2015;12(1):1–14. doi: 10.1186/s12985-015-0383-4 26411318 PMC4584467

[40] Hanley KA, Guerbois M, Kautz TF, Brown M, Whitehead SS, Weaver SC, et al. Infection dynamics of sylvatic dengue virus in a natural primate host, the African Green Monkey. Am J Trop Med Hyg. 2014;91(4):672–6. doi: 10.4269/ajtmh.13-0492 25092823 PMC4183386

[41] Castillo JA, Giraldo DM, Hernandez JC, Smit JM, Rodenhuis-Zybert IA, Urcuqui-Inchima S. Regulation of innate immune responses in macrophages differentiated in the presence of vitamin D and infected with dengue virus 2. PLoS Negl Trop Dis. 2021;15(10):e0009873. doi: 10.1371/journal.pntd.0009873 34634046 PMC8530315

[42] Valdés-lópez JF, Garcia GJF, Urcuqui-inchima S. Synergistic Effects of Toll-Like Receptor 1/2 and Toll-Like Receptor 3 Signaling Triggering Interleukin 27 Gene Expression in Chikungunya Virus- Infected Macrophages. 10.3389/fcell.2022.812110 PMC8863767 35223841

[43] Hernández-Sarmiento LJ, Tamayo-Molina YS, Valdés-López JF, Urcuqui-Inchima S. Mayaro virus infection elicits a robust pro-inflammatory and antiviral response in human macrophages. Acta Trop. 2024;252:107146. doi: 10.1016/j.actatropica.2024.107146 38342287

[44] Valdés-López JF, Fernandez GJ, Urcuqui-Inchima S. Interleukin 27 as an inducer of antiviral response against chikungunya virus infection in human macrophages. Cell Immunol. 2021;367:104411. doi: 10.1016/j.cellimm.2021.104411 34325085

[45] Kim D, Paggi JM, Park C, Bennett C, Salzberg SL. Graph-based genome alignment and genotyping with HISAT2 and HISAT-genotype. Nat Biotechnol. 2019;37(8):907–15. doi: 10.1038/s41587-019-0201-4 31375807 PMC7605509

[46] Pertea M, Pertea GM, Antonescu CM, Chang T-C, Mendell JT, Salzberg SL. StringTie enables improved reconstruction of a transcriptome from RNA-seq reads. Nat Biotechnol. 2015;33(3):290–5. doi: 10.1038/nbt.3122 25690850 PMC4643835

[47] Liao Y, Smyth GK, Shi W. featureCounts: an efficient general purpose program for assigning sequence reads to genomic features. Bioinformatics. 2014;30(7):923–30. doi: 10.1093/bioinformatics/btt656 24227677

[48] Zhang Y, Parmigiani G, Johnson WE. ComBat-seq: batch effect adjustment for RNA-seq count data. NAR Genom Bioinform. 2020;2(3):lqaa078. doi: 10.1093/nargab/lqaa078 33015620 PMC7518324

[49] Love MI, Huber W, Anders S. Moderated estimation of fold change and dispersion for RNA-seq data with DESeq2. Genome Biol. 2014;15(12):550. doi: 10.1186/s13059-014-0550-8 25516281 PMC4302049

[50] Valdés-López JF, Hernández-Sarmiento LJ, Tamayo-Molina YS, Velilla-Hernández PA, Rodenhuis-Zybert IA, Urcuqui-Inchima S. Interleukin 27, like interferons, activates JAK-STAT signaling and promotes pro-inflammatory and antiviral states that interfere with dengue and chikungunya viruses replication in human macrophages. Front Immunol. 2024;15(April):1–21. 10.3389/fimmu.2024.1385473 PMC11076713 38720890

[51] Yu G, Wang L-G, Han Y, He Q-Y. clusterProfiler: an R package for comparing biological themes among gene clusters. OMICS. 2012;16(5):284–7. doi: 10.1089/omi.2011.0118 22455463 PMC3339379

[52] Olagnier D, Peri S, Steel C, van Montfoort N, Chiang C, Beljanski V, et al. Cellular oxidative stress response controls the antiviral and apoptotic programs in dengue virus-infected dendritic cells. PLoS Pathog. 2014;10(12):e1004566. doi: 10.1371/journal.ppat.1004566 25521078 PMC4270780

[53] Maheshwari D, Saini K, Singh P, Singla M, Nayak K, Aggarwal C, et al. Contrasting behavior between the three human monocyte subsets in dengue pathophysiology. iScience. 2022;25(6):104384. doi: 10.1016/j.isci.2022.104384 35620424 PMC9127603

[54] Aguilar Briseño JA, Ramos Pereira L, van der Laan M, Pauzuolis M, Ter Ellen BM, Upasani V, et al. TLR2 axis on peripheral blood mononuclear cells regulates inflammatory responses to non-infectious immature dengue virus particles. PLoS Pathog. 2022;18(10):e1010499. doi: 10.1371/journal.ppat.1010499 36240261 PMC9605289

[55] Lai Y-C, Chao C-H, Yeh T-M. Roles of Macrophage Migration Inhibitory Factor in Dengue Pathogenesis: From Pathogenic Factor to Therapeutic Target. Microorganisms. 2020;8(6):891. doi: 10.3390/microorganisms8060891 32545679 PMC7356240

[56] Valdés-López JF, Fernandez GJ, Urcuqui-Inchima S. Synergistic Effects of Toll-Like Receptor 1/2 and Toll-Like Receptor 3 Signaling Triggering Interleukin 27 Gene Expression in Chikungunya Virus-Infected Macrophages. Front Cell Dev Biol. 2022;10:812110. doi: 10.3389/fcell.2022.812110 35223841 PMC8863767

[57] Song R, Gao Y, Dozmorov I, Malladi V, Saha I, McDaniel MM, et al. IRF1 governs the differential interferon-stimulated gene responses in human monocytes and macrophages by regulating chromatin accessibility. Cell Rep. 2021;34(12):108891. doi: 10.1016/j.celrep.2021.108891 33761354 PMC8300000

[58] Lester SN, Li K. Toll-like receptors in antiviral innate immunity. J Mol Biol. 2014;426(6):1246–64. doi: 10.1016/j.jmb.2013.11.024 24316048 PMC3943763

[59] Davis BK, Wen H, Ting JPY. The inflammasome NLRs in immunity, inflammation, and associated diseases. Annu Rev Immunol. 2011;29:707–35. 21219188 10.1146/annurev-immunol-031210-101405 PMC4067317

[60] Schroder K, Tschopp J. The inflammasomes. Cell. 2010;140(6):821–32. 20303873 10.1016/j.cell.2010.01.040

[61] Guglani L, Kabra SK. T cell immunopathogenesis of dengue virus infection. Dengue Bull. 2005;29:58–69.

[62] Coillard A, Segura E. In vivo Differentiation of Human Monocytes. Front Immunol. 2019;10:1907. doi: 10.3389/fimmu.2019.01907 31456804 PMC6700358

[63] Valdés-López JF, Urcuqui-Inchima S. Antiviral response and immunopathogenesis of interleukin 27 in COVID-19. Arch Virol. 2023;168(7). 10.1007/s00705-023-05792-9 PMC10261846 37310504

[64] Azeredo EL, Neves-Souza PC, Alvarenga AR, Reis SRNI, Torrentes-Carvalho A, Zagne S-MO, et al. Differential regulation of toll-like receptor-2, toll-like receptor-4, CD16 and human leucocyte antigen-DR on peripheral blood monocytes during mild and severe dengue fever. Immunology. 2010;130(2):202–16. doi: 10.1111/j.1365-2567.2009.03224.x 20113369 PMC2878465

[65] Malavige GN, Ogg GS. Pathogenesis of vascular leak in dengue virus infection. Immunology. 2017;151(3):261–9. doi: 10.1111/imm.12748 28437586 PMC5461104

[66] Kelley JF, Kaufusi PH, Nerurkar VR. Dengue hemorrhagic fever-associated immunomediators induced via maturation of dengue virus nonstructural 4B protein in monocytes modulate endothelial cell adhesion molecules and human microvascular endothelial cells permeability. Virology. 2012;422(2):326–37. doi: 10.1016/j.virol.2011.10.030 22129847 PMC3273497

[67] Green S, Vaughn DW, Kalayanarooj S, Nimmannitya S, Suntayakorn S, Nisalak A, et al. Elevated plasma interleukin-10 levels in acute dengue correlate with disease severity. J Med Virol. 1999;59(3):329–34. doi: 10.1002/(sici)1096-9071(199911)59:3<329::aid-jmv12>3.3.co;2-7 10502265

[68] Al Kadi M, Yamashita M, Shimojima M, Yoshikawa T, Ebihara H, Okuzaki D, et al. Cytokine storm and vascular leakage in severe dengue: insights from single-cell RNA profiling. Life Sci Alliance. 2025;8(6):e202403008. doi: 10.26508/lsa.202403008 40127923 PMC11933670

[69] Hsu Y-L, Wang M-Y, Ho L-J, Lai J-H. Dengue virus infection induces interferon-lambda1 to facilitate cell migration. Sci Rep. 2016;6:24530. doi: 10.1038/srep24530 27456172 PMC4960520

[70] Helbig KJ, Carr JM, Calvert JK, Wati S, Clarke JN, Eyre NS, et al. Viperin is induced following dengue virus type-2 (DENV-2) infection and has anti-viral actions requiring the C-terminal end of viperin. PLoS Negl Trop Dis. 2013;7(4):e2178. doi: 10.1371/journal.pntd.0002178 23638199 PMC3630087

[71] Dai J, Pan W, Wang P. ISG15 facilitates cellular antiviral response to dengue and west nile virus infection in vitro. Virol J. 2011;8:468. doi: 10.1186/1743-422X-8-468 21992229 PMC3215395

[72] Simon-Loriere E, Lin R-J, Kalayanarooj SM, Chuansumrit A, Casademont I, Lin S-Y, et al. High Anti-Dengue Virus Activity of the OAS Gene Family Is Associated With Increased Severity of Dengue. J Infect Dis. 2015;212(12):2011–20. doi: 10.1093/infdis/jiv321 26063222

[73] Dalrymple NA, Cimica V, Mackow ER. Dengue Virus NS Proteins Inhibit RIG-I/MAVS Signaling by Blocking TBK1/IRF3 Phosphorylation: Dengue Virus Serotype 1 NS4A Is a Unique Interferon-Regulating Virulence Determinant. mBio. 2015;6(3):e00553-15. doi: 10.1128/mBio.00553-15 25968648 PMC4436066

[74] Rodriguez-Madoz JR, Belicha-Villanueva A, Bernal-Rubio D, Ashour J, Ayllon J, Fernandez-Sesma A. Inhibition of the type I interferon response in human dendritic cells by dengue virus infection requires a catalytically active NS2B3 complex. J Virol. 2010;84(19):9760–74. doi: 10.1128/JVI.01051-10 20660196 PMC2937777

[75] Choi YH, Lim EJ, Kim SW, Moon YW, Park KS, An H-J. IL-27 enhances IL-15/IL-18-mediated activation of human natural killer cells. J Immunother Cancer. 2019;7(1):168. doi: 10.1186/s40425-019-0652-7 31277710 PMC6612093

[76] Gosselin J, TomoÏu A, Gallo RC, Flamand L. Interleukin-15 as an Activator of Natural Killer Cell-Mediated Antiviral Response. Blood. 1999;94(12):4210–9. doi: 10.1182/blood.v94.12.4210 10590066

[77] Pimentel JM, Zhou J-Y, Wu GS. The Role of TRAIL in Apoptosis and Immunosurveillance in Cancer. Cancers (Basel). 2023;15(10):2752. doi: 10.3390/cancers15102752 37345089 PMC10216286

[78] Schneider P, Mackay F, Steiner V, Hofmann K, Bodmer J, Holler N, et al. BAFF, a Novel Ligand of the Tumor Necrosis Factor Family, Stimulates B Cell Growth. J Exp Med. 1999;189(11):1747–56. 10359578 10.1084/jem.189.11.1747 PMC2193079

[79] Smulski CR, Eibel H. BAFF and BAFF-Receptor in B Cell Selection and Survival. Front Immunol. 2018;9:2285. doi: 10.3389/fimmu.2018.02285 30349534 PMC6186824

[80] Kohli K, Pillarisetty VG, Kim TS. Key chemokines direct migration of immune cells in solid tumors. Cancer Gene Ther. 2022;29(1):10–21. doi: 10.1038/s41417-021-00303-x 33603130 PMC8761573

[81] Palomino DCT, Marti LC. Chemokines and immunity. Einstein (Sao Paulo). 2015;13(3):469–73. doi: 10.1590/S1679-45082015RB3438 26466066 PMC4943798

[82] Hong H, Wu Y, Li Y, Han Y, Cao X, Wu VWY, et al. Endothelial PPARδ Ablation Exacerbates Vascular Hyperpermeability via STAT1/CXCL10 Signaling in Acute Lung Injury. Circ Res. 2025;136(7):735–51. doi: 10.1161/CIRCRESAHA.124.325855 39996324

[83] Rogers KJ, Maury W. The role of mononuclear phagocytes in Ebola virus infection. J Leukoc Biol. 2018;104(4):717–27. doi: 10.1002/JLB.4RI0518-183R 30095866

[84] Olejnik J, Forero A, Deflubé LR, Hume AJ, Manhart WA, Nishida A, et al. Ebolaviruses Associated with Differential Pathogenicity Induce Distinct Host Responses in Human Macrophages. J Virol. 2017;91(11):e00179-17. doi: 10.1128/JVI.00179-17 28331091 PMC5432886

[85] Gu Y, Zuo X, Zhang S, Ouyang Z, Jiang S, Wang F, et al. The Mechanism behind Influenza Virus Cytokine Storm. Viruses. 2021;13(7):1362. doi: 10.3390/v13071362 34372568 PMC8310017

[86] Vangeti S, Falck-Jones S, Yu M, Österberg B, Liu S, Asghar M, et al. Human influenza virus infection elicits distinct patterns of monocyte and dendritic cell mobilization in blood and the nasopharynx. Elife. 2023;12:e77345. doi: 10.7554/eLife.77345 36752598 PMC9977282

[87] Mulchandani R, Lyngdoh T, Kakkar AK. Deciphering the COVID-19 cytokine storm: Systematic review and meta-analysis. Eur J Clin Invest. 2021;51(1):e13429. doi: 10.1111/eci.13429 33058143 PMC7646004

[88] Del Valle DM, Kim-Schulze S, Huang H-H, Beckmann ND, Nirenberg S, Wang B, et al. An inflammatory cytokine signature predicts COVID-19 severity and survival. Nat Med. 2020;26(10):1636–43. doi: 10.1038/s41591-020-1051-9 32839624 PMC7869028

[89] Cong Y, McArthur MA, Cohen M, Jahrling PB, Janosko KB, Josleyn N, et al. Characterization of Yellow Fever Virus Infection of Human and Non-human Primate Antigen Presenting Cells and Their Interaction with CD4+ T Cells. PLoS Negl Trop Dis. 2016;10(5):e0004709. doi: 10.1371/journal.pntd.0004709 27191161 PMC4871483

[90] Melo JM, Falcão LFM, da Ponte LCT, Silva CC, Martins LC, Chiang JO, et al. Emergence of New Immunopathogenic Factors in Human Yellow Fever: Polarisation of the M1/M2 Macrophage Response in the Renal Parenchyma. Viruses. 2022;14(8):1725. doi: 10.3390/v14081725 36016347 PMC9416648

[91] Srivastava S, Dhoundiyal S, Kumar S, Kaur A, Khatib MN, Gaidhane S, et al. Yellow Fever: Global Impact, Epidemiology, Pathogenesis, and Integrated Prevention Approaches. Le Infez Med [Internet]. 2024;32(4):434–50. Available from: http://www.ncbi.nlm.nih.gov/pubmed/39660161%0A http://www.pubmedcentral.nih.gov/articlerender.fcgi?artid=PMC11627485 10.53854/liim-3204-3 PMC11627485 39660161

